# Micro- and nanofabrication of dynamic hydrogels with multichannel information

**DOI:** 10.1038/s41467-023-43921-9

**Published:** 2023-12-11

**Authors:** Mingchao Zhang, Yohan Lee, Zhiqiang Zheng, Muhammad Turab Ali Khan, Xianglong Lyu, Junghwan Byun, Harald Giessen, Metin Sitti

**Affiliations:** 1 Physical Intelligence Department, Max Planck Institute for Intelligent Systems, 70569 Stuttgart, Germany; 2 4th Physics Institute and Research Center SCoPE, University of Stuttgart, 70569 Stuttgart, Germany; 3 Institute for Biomedical Engineering, ETH Zürich, 8092 Zürich, Switzerland; 4 School of Medicine and College of Engineering, Koç University, 34450 Istanbul, Turkey

**Keywords:** Information storage, Gels and hydrogels, Gels and hydrogels

## Abstract

Creating micro/nanostructures containing multi-channel information within responsive hydrogels presents exciting opportunities for dynamically changing functionalities. However, fabricating these structures is immensely challenging due to the soft and dynamic nature of hydrogels, often resulting in unintended structural deformations or destruction. Here, we demonstrate that dehydrated hydrogels, treated by a programmable femtosecond laser, can allow for a robust fabrication of micro/nanostructures. The dehydration enhances the rigidity of the hydrogels and temporarily locks the dynamic behaviours, significantly promoting their structural integrity during the fabrication process. By utilizing versatile dosage domains of the femtosecond laser, we create micro-grooves on the hydrogel surface through the use of a high-dosage mode, while also altering the fluorescent intensity within the rest of the non-ablated areas via a low-dosage laser. In this way, we rationally design a pixel unit containing three-channel information: structural color, polarization state, and fluorescent intensity, and encode three complex image information sets into these channels. Distinct images at the same location were simultaneously printed onto the hydrogel, which can be observed individually under different imaging modes without cross-talk. Notably, the recovered dynamic responsiveness of the hydrogel enables a multi-information-encoded surface that can sequentially display different information as the temperature changes.

## Abstract (web summary)

Responsive hydrogels containing multichannel information have potential in miniature devices, but their fabrication can be challenging. Here, the authors report the creation of hydrogel materials with each pixel containing three-channel information for printing of distinct images in one location.

## Introduction

Micro/nanofabrication techniques have garnered significant attention across various fields, including integrated electronics, optics^1,2^, miniature robotics^3–6^, and microelectromechanical systems (MEMS)^7^. Among the tools employed for fabricating tiny structures^8,9^, femtosecond (fs) lasers stand out as highly efficient, versatile and extensively utilized strategies^10,11^. The distinguishing feature of fs lasers lies in their ability to deliver short pulse durations with exceptionally high peak intensity^12,13^. This characteristic facilitates precise energy deposition while minimizing heat transfer to the surrounding areas^14^. As a result, fs lasers empower the creation of high-precision structures across a wide spectrum of materials^11^. The versatility inherent in the photon-matter interactions achievable with fs lasers confers distinct advantages in micro/nanofabrication^12,15–17^. Processes such as photobleaching^17^, two-photon polymerization^11^, and multi-photon ablation^13^ enable tailored material modifications, substantially expanding the fabrication toolbox. Therefore, exploration and utilization of the diverse dosage domains offered by fs lasers opens up good possibilities in fabrication, presenting exciting opportunities for the integration of miniature devices across many applications.

However, despite the versatility of fs laser-based fabrication processes across a diverse range of materials, the creation of hydrogel micro/nanostructures poses a challenge^18,19^. Hydrogels, composed of three-dimensional (3D) hydrophilic polymer networks containing a high-water content (typically more than 90% by weight), have gained widespread attention owing to their versatile and easily tunable properties^20–22^. Nonetheless, the inherent softness of hydrogel materials, often characterized by low stiffness (typically on the order of kPa), presents significant hurdles in their high-precision fabrication^23,24^. The micro/nanostructures created within hydrogels often lack sufficient self-support, leading to structural instability and collapse^25^. Moreover, the inevitably generated thermal effects induced by the laser contribute to unwanted deformations of hydrogels, which arise either from the hydrogels’ thermoresponsive properties or the laser heating-induced bubbles, adding another layer of complexity to the precise fabrication of hydrogel structures^26,27^. These challenges compromise structural integrity, imposing formidable obstacles in achieving high-precision structures within hydrogels, restricting the potential applications of miniature devices fabricated from hydrogels.

Here, we introduce a general strategy that allows for the micro/nanofabrication within soft and dynamic hydrogels by dehydrating the hydrogel. The dehydration of the hydrogel not only bolster self-support of the fabricated structures but also transiently freezes their dynamic behaviours during the fabrication process. The versatility offered by various dosage domains of a fs laser empowers the creation of structures that can concurrently encompass multi-channel information within the responsive hydrogel, and exhibit multiple independent information at the same location on the hydrogel. Furthermore, we illustrate the dynamic capability of these structures in displaying intricate images sequentially through the thermal activation of the hydrogel.

## Results

### Micro/nanofabrication of multi-information within active hydrogels

**Fig. 1:**
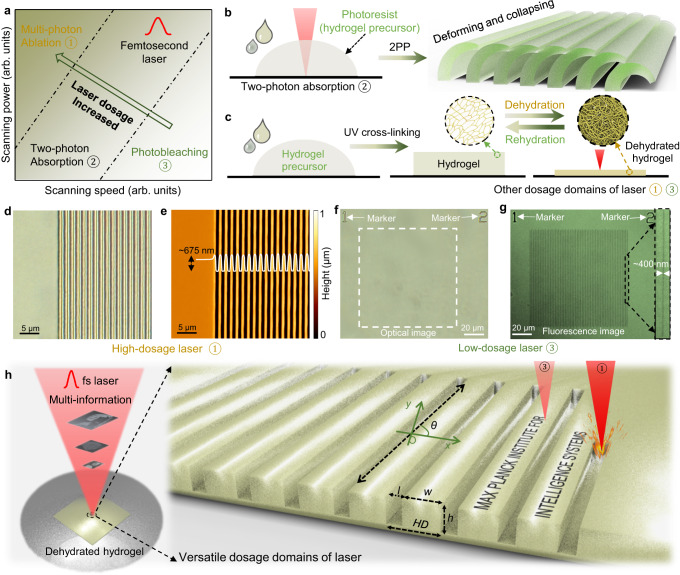
Dehydration of thermoresponsive hydrogels enabling their high-precision fabrication for encoding multi-information using multi-dosage of the femtosecond (fs) laser. **a** Versatile dosage domains of the fs laser and their different uses in the photon-material interactions. **b** Schematics showing the two-photon polymerized (2PP) hydrogels are prone to deform or collapse due to the low rigidity of hydrogels. **c** Schematic showing the dehydrated hydrogels enhance the structural integrity during their fs laser fabrication. **d** Optical image depicting the grooves on a hydrogel surface fabricated using laser with a high-dosage domain. **e** Atomic force microscopy (AFM) image showing these written grooves on the hydrogel surface. **f** Optical images illustrating a hydrogel surface using a low-dosage domain. The numbers “*1*” and “*2*” are abated as markers. **g** Confocal fluorescence microscopy image with hydrogel surface treated in the low-dosage domain. **h** Schematics of the micro/nanofabrication of the dehydrated hydrogel film with encoded multi-information.

While the versatility offered by fs lasers in enabling different photon-material interactions (Fig. 1a), the fabrication of intricate structures in soft hydrogels remains a formidable challenge. Hydrogel materials are known for their inherent softness (on the order of kPa). While the softness of hydrogels offers unique advantages, it also presents significant hurdles when it comes to creating micro/nanostructures. Numerous strategies have been attempted for micro/nanofabrication of hydrogels using fs lasers, including two-photon polymerization of hydrogel precursors (Fig. 1b and Supplementary Fig. 1a), hydrogel molding (Supplementary Fig. 1b), and direct laser ablation of hydrogels (Supplementary Fig. 1c). Unfortunately, these approaches have fallen short in achieving high-precision micro/nanostructures with high structural integrity in hydrogels (see detailed discussion in Supplementary Note 1). We propose a method to bolster their mechanical rigidity that relies on a reversible dehydration process of hydrogels (Fig. 1c and Supplementary Fig. 2). This process results in the compaction of the original porous network within the hydrogel, rendering a substantial enhancement in mechanical properties. Furthermore, the dynamic behaviour of hydrogels can be temporarily arrested or “frozen”, contributing to an augmented structural integrity during the fs laser fabrication process. These dehydrated hydrogels, with intricately fabricated structures by fs laser, can recover their original porous network (high water content) and stimuli-response through rehydrating in water.

By rationally programming the delivered dosage (fluence, the delivered energy per unit area) of the laser for versatile photon-matter interactions, we can enable the micro/nanofabrication of a thermal-responsive hydrogel, which we recently developed^28^ (LIHAM, Supplementary Fig. 3), and encode diverse multi-information. A high dosage of the laser makes it possible to write uniform grooves (i.e., surface relief gratings) with submicron resolution on the surface of the dehydrated hydrogels (Fig. 1d, e), while a low dosage laser can induce notable changes to the intrinsic chemical information within the hydrogels (Fig. 1f, g). For example, exposing a fluorescent dye (like phenol red) inside the hydrogel to a low-dosage domain of the laser can cause the dye to photobleach, leading to a decrease in its fluorescent intensity. As shown in the optical image in Fig. 1f, no physical damage could be seen on the surface, while the confocal fluorescence image revealed that arrays of dark lines had been already written due to the bleaching (Fig. 1g). These multiple channels of information can be designed to decouple the various types of information encoded (Fig. 1h). Furthermore, since the rehydrated LIHAM films are thermally-responsive, the structures written on them can be dynamically tuned, providing a stimuli-responsive means of manipulating the encoded information.

### Optimization of the fabricated structures

**Fig. 2:**
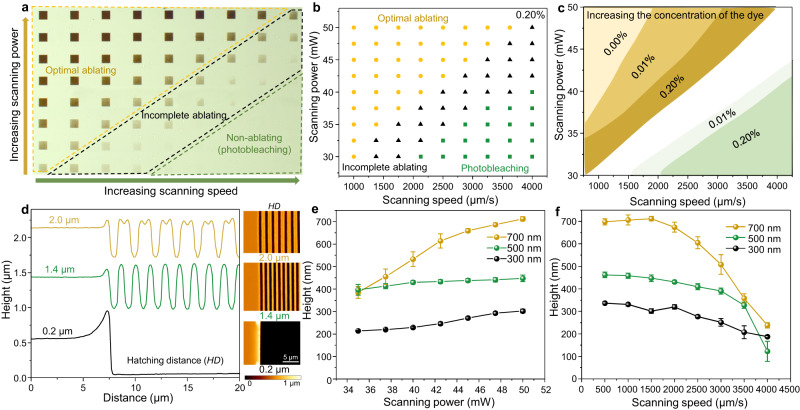
Optimization of the fabricated structures by changing the concentration of photothermal dyes in the hydrogel film and tuning scanning parameters of the fs laser. **a** Optical image depicting written squares (composed of with a grating length of 100 µm and a hatching distance (*HD*) of 1.5 µm) with different scanning powers and speeds. **b** Chart displaying three written domains of a hydrogel film with 0.2 wt% dopant of phenol red (photothermal dye): optimal ablation, incomplete ablation, and photobleaching domain. **c** Effect of the concentration of the phenol red in the hydrogel film on the written ablation and photobleaching domains. **d** Topologies of three typical grooves on the hydrogel surface with different *HDs* measured by AFM*.* **e** Effect of the scanning power of the laser on the height of the written grooves of three hydrogel films with different thicknesses. Data points are shown as mean ± s.d. (*n* = 14). **f** Effect of the scanning speed on the height of written grooves of three hydrogel films with different thicknesses. Data points are shown as mean ± s.d. (*n* = 14).

To ensure the precise and complete encoding of different information onto the dehydrated LIHAM film, it is crucial to optimize the ablated grooves, which serve as the basic building blocks. This can be achieved by manipulating different dosage domains of the laser through the tuning of the scanning speeds and powers of lasers, which can result in three written domains: optimal ablation, incomplete ablation, and photobleaching domains (Fig. 2a). When the scanning power is increased or the scanning speed is decreased, higher doses of the laser are addressed onto the LIHAM film, making ablation more likely to occur (Fig. 2b). It is important to avoid incomplete ablation domains, as they contain incomplete or coupled information from both ablation and photobleaching. By doping a fluorescent dye (phenol red), the photothermal efficiency can be improved, which can adjust the boundaries of the different laser dosage domains required for multi-photon ablation and photobleaching. A small doping amount (0.2 wt%) of phenol red can significantly shift the ablation domain to a wider lower-dosage range (i.e., increase the range of scanning powers and speeds) and also decrease the dosage required for photobleaching of the fluorescent dye (Fig. 2c and Supplementary Fig. 4, further optimization of photobleaching is discussed in the *programmable photobleaching of the fluorescent dye* section).

The geometries of the ablated grooves can be optimized by controlling the printing parameters of the fs laser, such as hatching distance (*HD*), scanning powers, and scanning speeds. It is important to ensure that the value of *HD* exceeds the length (*w*) of the individual grating; otherwise, the hydrogel may receive an overdose of the laser radiation, causing complete ablation of certain areas (e.g., *HD* = 0.2 µm, as indicated in Fig. 2d). When *HD* ≥ 1.0 µm, distinguishable grooves can be created. It should be noted that the heights between neighboring grooves are higher than the initial surface level due to the thermal stress induced by the laser during ablation, causing the hydrogel to expand to both edges and form peaks above the initial level (e.g., *HD* = 1.4 µm). Increasing *HD* further can result in the formation of two peaks between adjacent grooves (e.g., *HD* = 2.0 µm). In the following study, *HD* was set to 1.5 µm, unless otherwise specified. Additionally, scanning powers and speeds can be adjusted to tune the topological morphologies of the grooves (as suggested in Fig. 2a). Modifying the dosage of laser by increasing the scanning power or reducing the scanning speed can tailor the morphologies of the obtained grooves and result in the creation of a deeper groove (Fig. 2e, f) and a broader full width at half maximum (FWHM) of the groove (Supplementary Fig. 5). Furthermore, the tunability of the height varies depending on the thickness of the initial films, with thicker films tending to form wider tunable heights (Fig. 2f).

### Programmable colors of the ablated grooves

**Fig. 3:**
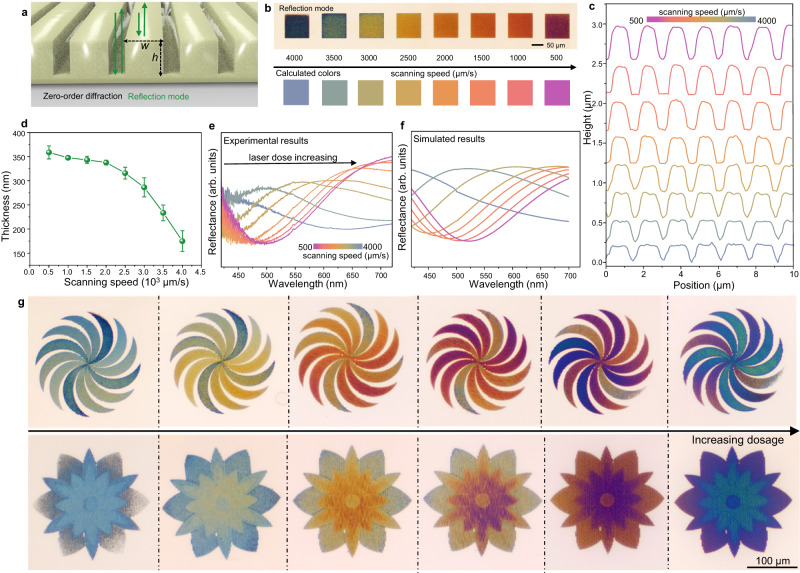
Programmable color of the grooves with tunable heights. **a** Schematics of the gratings showing color generation in the reflection mode. Important parameters include width (*w*) and height (*h*) of the gratings. **b** Optical images of the written squares (with a scanning power of 50 mW) in the reflection mode, with calculated colors from the correspondingly measured reflection spectra. **c** Profiles of the grooves upon different scanning speeds measured by AFM. **d** Effect of the scanning speeds on the heights of the obtained grooves. Data points are shown as mean ± s.d. (*n* = 4). **e** Experimental reflection spectra of the grooves fabricated with different scanning speeds. **f** Simulated reflection spectra of the corresponding grooves. **g** Demonstrations of the colorful patterns with programmable colors. The scanning speed is set at 1000 µm/s, while the scanning power varies within a range of 30-50 mW at different locations within the written patterns.

The rationally engineered grooves obtained on the LIHAM surface show tunable optical spectra in the visible range and can thus generate programmable colors^29^. These grooves can be analyzed as a periodic dielectric grating with a periodicity (*HD*) of 1.5 µm (refer to Fig. 3a). The reflectance spectra of such structures are determined by the presence of optical resonance modes within specific wavelength regions. When light is confined within the LIHAM cavity, it undergoes multiple reflections, resulting in the formation of modes with specific resonance wavelengths. The thickness (*h*) of the LIHAM structure determines the wavelengths at which these resonances occur, creating standing wave modes within the structure (Supplementary Fig. 6a). Additionally, the width (*w*) of the grating can also affect the resonance phenomenon (Supplementary Fig. 6b). In this study, diffraction occurs as a result of the grating’s period being larger than the wavelength of the visible range. However, the higher order diffraction components can be disregarded due to their negligible power compared to that of the zero-order diffraction (Supplementary Fig. 7). Furthermore, considering the small numerical aperture (NA) of the objective (0.13), the transmitted and reflected light in the visible range primarily consists of the contribution from zero-order diffraction, as described by the following equation: $\theta={\sin }^{-1}\frac{m\lambda }{\Lambda }$, where *m* denotes the order of diffraction, *λ* is the incident wavelength, and *Λ* is the period of the grating.

As stated above, the heights of the grooves can be programmed by adjusting the scanning parameters, which enables control over the observed colors of the groove-based gratings (Fig. 3b). For instance, varying the scanning speed of the laser (500–4000 µm/s) can result in different *h* (170–360 nm, Fig. 3c, d) of the grooves and *w* of the grating, which results in tunable peaks in the measured optical spectra (Supplementary Fig. 8). Various colors, plotted in a CIE1991 chromaticity diagram (Supplementary Fig. 9), are successfully demonstrated with different heights (addressed by the laser with different scanning speeds), and their corresponding reflectance spectra show good agreement with simulated spectra (Fig. 3e, f). Thus, direct writing of patterns with arbitrary colors on the LIHAM surface can be achieved by rationally designing the scanning powers and/or speeds. For example, patterns of a windmill and a flower were written with different scanning powers in each part (using the same scanning speed, Supplementary Fig. 10), producing multiple colors within these individual patterns (Fig. 3g), and a wide range of colors could be realized by varying the scanning speeds.

### Programmable polarization of the ablated grooves

**Fig. 4:**
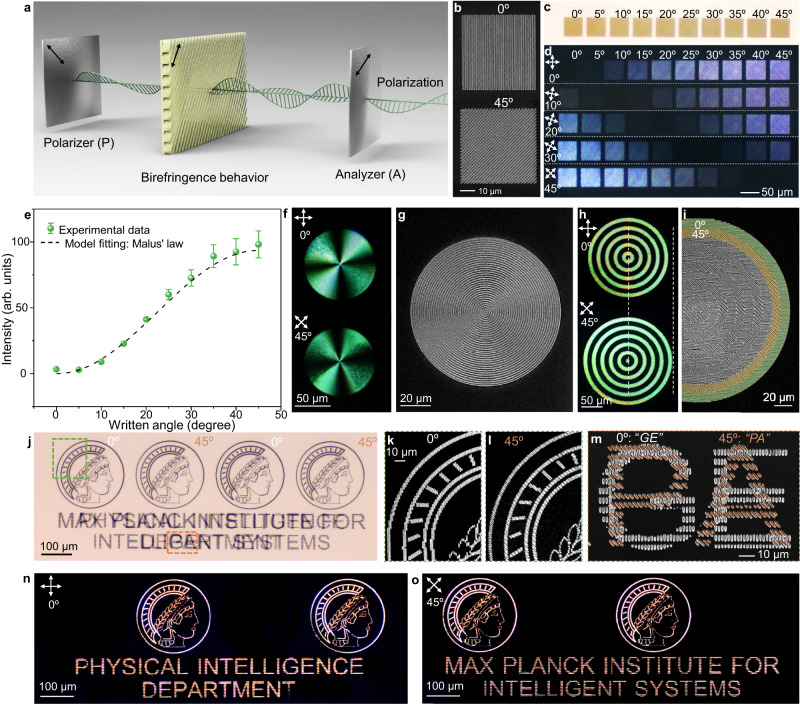
Programmable light-intensity of the groove-based gratings with tunable orientations. **a** Schematics depicting the birefringence behaviour observed from the oriented gratings. **b** Scanning electron microscopy (SEM) images displaying two typical gratings with written angles of 0 and 45 degrees. **c**, **d** Optical (**c**) and cross-polarized (**d**) images of square gratings with different written angles. **e** Light intensity of the square grating with different written angles and the fit according to Malus’ law. Data points are shown as mean ± s.d. (*n* = 12). **f** Cross-polarized images with a concentric pattern under 0° and 45° rotation angles. **g** SEM image of the concentric pattern. **h** Cross-polarized images with a concentric-belt pattern with alternatively writing angles of 0° and 45° under 0° and 45° rotation angles. **i** SEM image of the concentric-belt pattern. **j** Reflective optical image showing dual-information. **k**, **l** SEM images of the zoom-in logo of *Max Planck Institute* with written angles of 0° (**k**) and 45° (**l**). **m** SEM image of the zoom-in overlapped letters with written angles of 0° for the two letters of “*GE*” and 45° for another two letters of “*PA*”. **n**, **o** Cross-polarized optical images of the dual-information with written angles of 0° (**n**) and 45° (**o**). The scanning speed and power are set at 1000 µm/s and 50 mW for writing the above samples.

These groove-based gratings not only produce vivid colors but also exhibit remarkable birefringence behaviour. The spatial asymmetry of these grooves brings about varying reflective indices along these gratings, endowing them with substantial optical anisotropy, which can be utilized to program variable light-intensity under a cross-polarized linear light source^28,30^. When a polarized light passes through these gratings, it is refracted into two orthogonal rays: one along and one perpendicular to the groove orientation. These rays travel at different velocities due to the varying refractive indices along the grating. Consequently, only components of the two rays along the analyzer acquire a certain phase difference (Fig. 4a). Interestingly, a series of square gratings with different orientations (Fig. 4b, c) did not exhibit any noticeable difference without polarizers in their reflective optical images. However, their light intensities increased incrementally from total darkness (extinction) to the maximum brightness as their written orientations increased from 0° to 45° under the cross-polarized images. Moreover, rotating the stage of the square gratings resulted in the alternating shift of their light intensities (Fig. 4d). The orientation-dependent light-intensity of these gratings agrees with the Malus’ law^31^ (Fig. 4e).

The birefringence behaviour of the written grating provides a basis for angle-dependent information display. By manipulating the written angles of the grating-based pixel, we can locally control the display intensity of each pixel, and achieve the construction of complex information. For instance, a pattern composed of concentric grooves under cross-polarized light showed typical four bright/dark brushes, and rotating the plate did not change the relative positions of the alternative brushes (Fig. 4f). This can be understood that concentric grooves are rotational symmetry, and the 0°/90° textures along the orientation of the polarizer and analyzer remain unchanged regardless of the rotation of the stage (Fig. 4g). Similarly, a pattern consisting of concentric belts with alternatively written angles of 0°/45° displayed a series of spaced circle belts (Fig. 4h, i). These adjacent bright and dark concentric belts succeed each other after every 45° rotation of the stage. Moreover, we further realized dual-information displaying by rationally designing the overlapped angle-dependent information (Fig. 4j). The pattern consisted of four logos of Max-Planck Institute with alternative written angles of 0°/45° (Fig. 4k, l). Additionally, the textual information of “*Physical Intelligence Department*” (45°) and “Max-*Planck Institute for Intelligent Systems*” (0°) were mutually spaced (Fig. 4m). As a result, these logos alternately emerge when rotating the stage (Supplementary Movie 1). The textual information of “*Physical Intelligence Department*” can be read when placed at an angle of 0° (Fig. 4n), and that of “*Max-Planck Institute for Intelligent Systems*” appears after another 45° rotation (Fig. 4o).

### Programmable photobleaching of the fluorescent dye

**Fig. 5:**
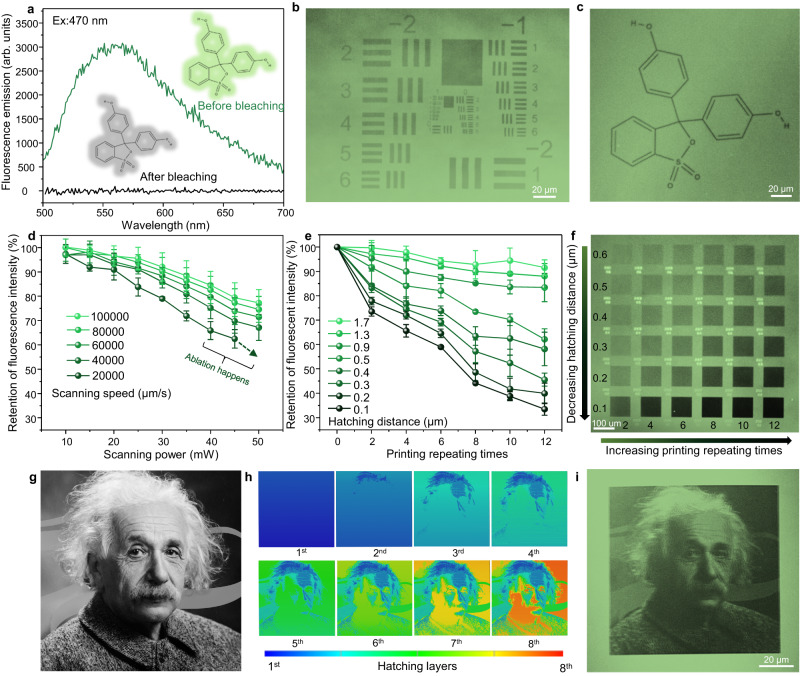
Programmable photobleaching of the fluorescent dye. **a** Fluorescence emission spectra of the LIHAM film before and after the laser treatment (excited with a source of 470 nm). **b** Fluorescent microscopy image of the USAF 1951 resolution chart (192-kDa PS/BDABP) demonstrating the written resolution on the LIHAM surface. **c** Fluorescent microscopy image depicting a written pattern of the chemical formula of Phenol red. **d** Effect of the scanning speeds and powers on the retention of fluorescence intensity. Data points are shown as mean ± s.d. (*n* = 14). **e** Effect of *HDs* and hatching layers on the retention of fluorescence intensity. Data points are shown as mean ± s.d. (*n* = 14). **f** Fluorescent microscopy image displaying written squares with different *HDs* and hatching layers. **g** Photograph of *Albert Einstein*. **h** Simulation of the writing process for the image of *Albert Einstein*. **i** Fluorescent microscopy image of the written images. The scanning speed and power are set at 100,000 µm/s and 50 mW for writing the above samples.

In addition to using the height and orientation of the ablated grooves to store information, we discovered that we could also use the areas between grooves as an additional channel for information writing. By using a low-dosage laser, we could modify the chemical information and cause photobleaching inside these areas to create additional patterns^32^. We found that the dye phenol red inside the LIHAM emitted light at around 560 nm, and that its emission disappeared after being treated with a low-dosage fs laser (Fig. 5a). By adjusting various parameters such as scanning power, scanning speed, *HD*, and hatching layer, we are able to achieve a written resolution down to the submicron scale using a USAF1951 resolution target (Figs. 1e and 5b). Using this method, we could create versatile patterns on the LIHAM surface (Fig. 5c).

We also explored the direct writing of full grayscale images, which required studying the controllable photobleaching of the dye. We found that reducing the scanning speed or increasing the scanning power causes a higher degree of photobleaching (Fig. 5d), but this is limited by the risk of unwanted ablation to the LIHAM surface (Supplementary Fig. 11). Instead, we increased the number of written repeats (hatching layer) with a rather mild dosage, which allows us to program photobleaching without causing ablation (Fig. 5e, f). Additionally, a decreased *HD* leads to an expansion of the photobleached area, ultimately causing a remarkable decrease in the overall fluorescent intensity (Fig. 5e, f). Unless otherwise stated, we typically use *HD* = 0.2 µm for the photobleaching process. By taking advantage of the good tunability of photobleaching, we were able to write complex grayscale images on the LIHAM surface (Fig. 5g). By projecting the grayscale pixels (0-255) of the image onto a certain number of layers, we could encode the image information in a layer-by-layer fashion (Fig. 5h), and finally achieve the direct writing of complex images, such as an image of *Albert Einstein* (Fig. 5i).

### Integration of multi-channel information within hdyrogels for dynamic information display

**Fig. 6:**
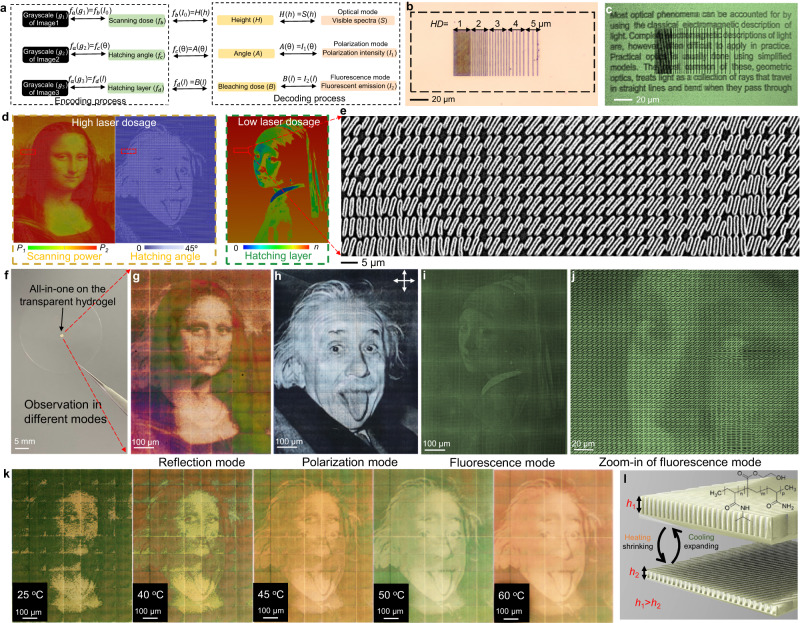
Direct writing of multi-channel information on the LIHAM surface via programming laser dosage domain of fs laser and its dynamic information display. **a** Chart illustrating the encoding and decoding process of multi-channel information. **b** Reflective optical image of the LIHAM surface written by ablated lines with different *HD* and photobleached text behind. **c** Fluorescent microscopy image of the LIHAM surface written with ablated lines with different *HD* and photobleached text. **d** Simulation of the printing process of multi-channel information, including high-dosage domain (hatching powers and scanning powers) and low-dosage domain (hatching layers) of the fs laser. **e** SEM images of the morphologies of a written surface. **f** Photograph showing the all-in-one information written on the transparent LIHAM hydrogel. **g** Colorful image *Mona Lisa* observed in a reflective optical microscope. **h** Grayscale image *Albert Einstein* observed in a cross-polarized optical microscope. **i** Grayscale image showing *Girl with a Pearl Earring* observed in a confocal fluorescent microscope. **j** Zoom-in fluorescent image of the *Girl with a Pearl Earring* image. **k** Reflective optical images of the observed Mona Lisa image shifting to Albert Einstein image as the temperature changes from 25 to 60 °C. **l** Schematics showing the height deformation (*h*_1_ to *h*_2_) of the grating structures of the LIHAM surface as a result of temperature change.

The geometry parameters of grooves and the fluorescent dye between two adjacent grooves can provide three channels for writing multi-channel information (Fig. 6a). Taking the grooves as the building pixels, we can map the grayscale values of the first and second images into sets of scanning powers (scanning dosage) and hatching angles for individual pixels, and the heights and orientations of the pixels as two channels can thus be encoded with the two sets. The third image (fluorescent information) can be mapped into different hatching layers, and encoded into the LIHAM surface with the low-dosage laser.

To integrate multiple information channels and write directly onto the LIHAM surface without causing cross talk, parameters of the grating unit need to be optimized. The ablated grooves tend to fully erase the fluorescent information behind, and thus the *HD* of the grooves has a large effect on the writable availability of the fluorescent channel (Supplementary Fig. 12). As illustrated in Fig. 6b, c, textual information behind these hatching lines will be largely retained as the *HD* increases. Additionally, as the scanning powers and speeds of the laser for ablating grooves can cause different geometries to the ablated grooves, they thus leave different availability among the unablated areas for writing fluorescent information (Supplementary Fig. 13). Therefore, a rational design of parameters of the laser determing the density of grooves in each pixel can provide a balance loading for addressing different information. By programming each pixel (with a size of 5 × 5 µm^2^) with integrated parameters of scanning power, hatching angle, and hatching laser (Fig. 6d), three different images can be written onto the same site of the LIHAM surface (Fig. 6e). These images written on the hydrogel surface (Fig. 6f) can be directly observed under an optical microscope, a cross-polarized optical microscope, and a fluorescent microscope, respectively. A colorful image of *Mona Lisa* in reflective mode (Fig. 6g), and a grayscale image of *Albert Einstein* in polarized mode (Fig. 6h), and a grayscale image of *Girl with a Pearl Earring* in fluorescent mode (Fig. 6i, j), can be seen, without apparent “cross-talk” effect between the channels.

Notably, the rehydrated hydrogel used in this study exhibits large isotropic shape changes in response to temperature, enabling the encoding of dynamic and changing information. An intriguing example involves gradually increasing the temperature from 25 to 60 °C, causing the image of *Mona Lisa* (encoded in the heights of grooves) observed in reflection to vanish gradually and be replaced by the image of *Albert Einstein* (encoded in the angles of grooves) (Fig. 6k and Supplementary Movie 2). Although the exact mechanism behind this switching between different channels still requires further investigation, it is reasonable to hypothesize that the changes in groove heights under heating play a significant role in this switching process (Fig. 6l). As demonstrated by the grating heights measured by in-situ AFM, they decrease when the temperature rises (Supplementary Fig. 14). This height decrease is attributed to the hydrophilic-to-hydrophobic transition of the LIHAM, which gradually releases internal water as the temperature surpasses the lower critical solution temperature (LCST). To further understand the influence of groove height on image switching, we fabricated different samples with varied height ranges. By sequentially increasing the height ranges of the written gratings through enhanced laser dosage (decreased scanning speeds) during each writing process, the individual image observed in reflection gradually evolves from *Albert Einstein* to *Mona Lisa* (Supplementary Fig. 15). Furthermore, it is worth noting that the thermal deformation of the LIHAM hydrogel is fully reversible^28^, and the information encoded within the hydrogel thus re-emerges upon cooling. This controllable increase in the height range provides insight into the observation of images switching between different channels, as the thermal actuation of the LIHAM in water leads to the shrinkage of the grating heights. This fascinating effect enables the switching between different images and the dynamic display of information at the same location on the LIHAM surface, providing exciting opportunities for applications in encryption, camouflage, information storage, and smart displays.

## Discussion

While the softness of hydrogels offers distinct advantages, it also poses significant challenges when it comes to creating micro/nanostructures. In our work, a general strategy of hydrogel dehydration has been implemented before fs laser fabrication, which significantly increases the structural integrity of the resulting micro/nanostructures. Within these, we have harnessed versatile dosage domains of fs laser and successfully achieved the direct writing of multi-channel information within thermally-responsive hydrogels. The high-dosage mode of the laser is utilized to create submicron grooves on the hydrogel surface, while we employ a low-dosage laser to modify the fluorescent intensity in the unablated areas between two adjacent grooves. By rationally programming the laser using these versatile dosage domains, we are able to impart a basic unit with three distinct channels of information: structural color, polarization state, and fluorescent intensity. This approach using versatile dosage of laser allows us to write three different images at the same location on the hydrogel surface, with each image observable individually under different modes without interference. Furthermore, the micro/nanostructures we have created within the hydrogels currently contain only two-dimensional (2D) multi-information. Nevertheless, we envision that our approach can be extended to fabricate intricate structures that include 3D information, such as 3D polarization structures of metasurfaces^33^, and this expansion will provide exciting possibilities for achieving even more sophisticated functionalities and applications. Additionally, since the hydrogel is thermally responsive after rehydration, the multi-information-encoded surface can sequentially display different information as the temperature changes. This opens up captivating possibilities for dynamic and reconfigurable functionalities offered by various functional materials.

## Methods

### Synthesis of the LIHAMs

The precursor solution for making the LIHAM was prepared by dissolving N-Isopropylacrylamide (monomer, NIPAM, Sigma-Aldrich, ≥99%), acrylamide (monomer, AM, Sigma-Aldrich, ≥99%), Hydroxyethyl methacrylate (monomer, HEMA, Sigma-Aldrich, ≥97%), Lithium phenyl-2,4,6-trimethylbenzoylphosphinate (photoinitiator, TPO-Li, Sigma-Aldrich, ≥95%), Poly(ethylene glycol) diacrylate (cross-linker, PEGDA, Sigma-Aldrich, Mn 575) into DI water at a weight ratio of 500: 30: 30: 10: 150: 4500 (NIPAM: AM: HEMA: TPO-Li: PEGDA: DI water). Phenol red was added to enhance the photothermal efficiency of the LIHAM, and thus the dosage domain of the laser could be tuned during the writing optimization. Additionally, it served as another channel for encoding information through controllable photobleaching.

Capillary cells with different thicknesses were assembled on round glass substrates using silica balls (MicroParticles GmbH) as spacers. By filtrating hydrogel precursor into the capillary cells and cross-linking with 365 nm UV light for 15 min, different thicknesses of LIHAM film was obtained on the glass substrate after opening the cover glass. The LIHAM film was dehydrated in an ambient environment for 3 hours, facilitating a complete water evaporation. Thicker (or bulk) LIHAM samples require an extended drying process in a vacuum drying oven at 80 °C for 5 hours to ensure thorough drying.

### Micro/nanofabrication of multi-information within LIHAM Hydrogels

The dehydrated LIHAM film on the glass substrate was then directly written through the fs laser (center wavelength: 780 nm, pulse duration: 80 fs, repetition rate: 80 MHz, average output power (scanning power): 0–50 mW) with programmable dosage domains and writing processes (DeScribe software, Nanoscribe GmbH). We used a 63X oil-immersion objective (NA = 1.4, focal width (FWD) = 0.19 mm, parfocal length = 45.06 mm, Zeiss) to enable the micro/nanofabrication within the LIHAM film. The laser dosage was programmable, allowing for the creation of different dosage domains by adjusting parameters such as scanning power, scanning speed, hatching distance, and hatching laser. The high dosage of the laser was used for ablation, while the low dosage was used for the photobleaching of fluorescence molecules. The writing codes integrating three-channels information include information of the grayscale values of three images (0–255), which were converted into the mappings of scanning power (with a range of 15–50 mW) set for image 1, hatching direction (with a range of 0–45°) set for image 2, and the mapping of hatching layers (with a range of 0–10 layers, image 3) set for image 3. The scanning speed is set at 1500 µm/s for writing images 1 and 2; the scanning speed and power are set at 100,000 µm/s and 30 mW for writing image 3. These mappings were then combined to create an all-in-one writing code. By applying the integrated code in the Nanoscribe system, we could directly write multi-information at the same location of the LIHAM surface. The as-fabricated samples were coated with a 20 nm Au layer to enhance their reflection intensity. The as-fabricated samples could be viewed under different microscope modes, including reflective optical microscope, cross-polarized microscope, and fluorescent microscope. For dynamic behaviour observation, the written sample would need to be sealed into a water chamber and mounted into an optical heater.

### Characterization

An optical microscope (Zeiss; Axio Imager 2) with reflection and cross-polarized modes was used to observe the written information on the LIHAM surface. An atomic force microscope (NanoWizard 4, JPK Instruments) was used to measure the height of the ablated grooves, and the dynamical change in the height of the ablated grooves was measured by the AFM equipped with an in-situ heating plate. The morphology of the ablated groove structures was measured by a scanning electron microscope (Leo Gemini 1530). The fluorescent information of the written sample was measured by a confocal fluorescent microscope (SP8, Leica). The spectra of the written micro-pixels were recorded by a custom-built microspectroscopy setup based on a Princeton Instruments grating spectrometer (SP2500i) with a Peltier-cooled CCD camera (PIXIS 256E). The fluorescence emission spectra of the LIHAM before and after the laser treatment were measured by a plate reader (BioTek Synergy 2 Microplate Reader). The compression tests of the LIHAM hydrogel in fully swollen and dehydrated states were measured by a hybrid rheometer (Discovery HR-3, TA instruments).

### Simulation of the reflection spectra

Rigorous coupled wave analysis (RCWA), a kind of Fourier modal method for full electromagnetic field simulation used to simulate the reflection spectra of the fabricated gratings. For two-dimensional simulation, the truncation order related to the size of Fourier coefficient matrix was set to 130 since the simulation results converge consistently when the truncation order was greater than 80. In our calculation, the refractive indices of LIHAM (dry film) and substrate were set as 1.45 and 1.46.

### Supplementary information

Supplementary Information

Peer Review File

Description of Additional Supplementary Files

Supplementary Movie 1

Supplementary Movie 2

### Source data

Source Data

## Data Availability

The data needed to evaluate the conclusions in this work are publicly available online. Additional data related to this paper may be requested from the corresponding authors upon request. Source data are provided with this paper.
