# Supplementary material for: Micro- and nanofabrication of dynamic hydrogels with multichannel information
Source: Nat Commun. 2023 Dec 11;14:8208. doi: 10.1038/s41467-023-43921-9 (PMC10713606; doi:10.1038/s41467-023-43921-9)
Supplement: Supplementary file 1 — Supplementary Information [file 41467_2023_43921_MOESM1_ESM.pdf]

Supplementary Information for

Nano- and microfabrication of dynamic hydrogels with multi-channel information

Mingchao Zhang, Yohan Lee, Zhiqiang Zheng, Muhammad Turab Ali Khan, Xianglong Lyu, Junghwan Byun, Harald Giessen, Metin Sitti*

This PDF file includes:

Supplementary Note 1
Supplementary Figs. 1 to 15
Supplementary References 1 to 7

Supplementary Note 1. Typical stragies using femtosecond laser for the micro- and nanofabrication of hydrogel materials

Femtosecond (fs) lasers have emerged as a highly efficient and versatile strategy for fabricating micro- and nanostructures across a wide range of materials[1]. This technique leverages versatile photon-matter interactions and encompasses both additive and subtractive manufacturing approaches.

Additive Manufacturing: One notable additive manufacturing approach for creating high-precision hydrogels is known as two-photon polymerization (2PP)[2,3]. In this process, a hydrogel precursor containing dispersed monomers in a solvent, typically water, undergoes site-specific crosslinking through two-photon absorption induced by the fs laser. This results in the creation of high-precision 3D structures at submicron scales (see Supplementary Fig. 1a). However, hydrogel structures produced through 2PP, especially those with high aspect ratios, are susceptible to deformation or collapse due to their low stiffness[4]. This is primarily a consequence of their high water content (exceeding 90%). While solvent-free hydrogel precursors can improve structural integrity due to their stiff 3D polymer structures[5], this also leads to much higher cross-linking density of networks. As a consequence, the later water absorption is limited due to the high cross-linking density, resulting in a departure from typical hydrogel properties like softness and responsiveness based on water adsorption/desorption, which are permanently compromised.

Another additive manufacturing strategy involves molding precision structures from a 2PP-fabricated master mold[6]. Hydrogel precursor solution is cast onto the master mold, cross-linked with UV light, and then detached (Supplementary Fig. 1b). However, this method is also hampered by the softness of hydrogel materials, leading to problems like line/plane defects and cracks during demolding process due to the fragile and soft nature of hydrogels.

Subtractive Manufacturing: Subtractive manufacturing techniques involve directly exposing materials to fs laser ablation, allowing for the creation of high-precision structures. Nevertheless, the high water content and dynamic behavior of hydrogels pose significant challenges to achieving precision in this process. Laser-induced heating leads to intense water evaporation (generating disturbing bubbles) within the

hydrogel polymer networks, causing deformation and defects during ablation. Additionally, certain hydrogels, such as thermally responsive variants, are sensitive to temperature changes, further undermining structural integrity.

Our Dehydration-Based Strategy: As structures made of most commercial photoresits are usually rigid (usually with moduli on the order of GPa), they can achieve high-precision farbication. Therefore, we believe that as materials become increasingly rigid, the fabricated high-precision structures tend to become more self-supporting, thereby enhancing the structural integrity of the resulting micro/nanostructures (Supplementary Fig. 2a). Therefore, it can be advantageous to improve their mechanical properties by dehydrating the hydrogels before laser processing, as illustrated in Supplementary Fig. 2b. The dehydration process serves a dual purpose: it not only enhances the mechanical properties (Young's modulus) of hydrogels more than three orders of magnitude (Supplementary Fig. 2c), but also temporarily stabilizes their dynamic behavior. The reason behind this effect lies in the transformation of the porous 3D network of hydrogels into a more compact structure after dehydration (Supplementary Figs. 2d and 2e). The stabilization is particularly beneficial for responsive hydrogels, such as the one we have recently developed (LIHAM)[7]. As the thermal response of these hydrogels is governed by the desorption/adsorption of water, the dehydration process (absence of water) can effectively arrest or freeze their thermal response.

Importantly, this dehydration does not alter the cross-linking density of the hydrogel. As a result, the dehydrated hydrogel can recover its high water content after micro/nanofabrication, thus retaining its thermal responsiveness. In this way, this reversible dehydration/rehydration process enables dynamic and reconfigurable functionalities in the fabricated structures. Our strategy offers a solution to the challenges associated with precision hydrogel manufacturing using fs lasers, opening up new possibilities for the dynamic and responsive design of hydrogel-based miniature structures and devices.

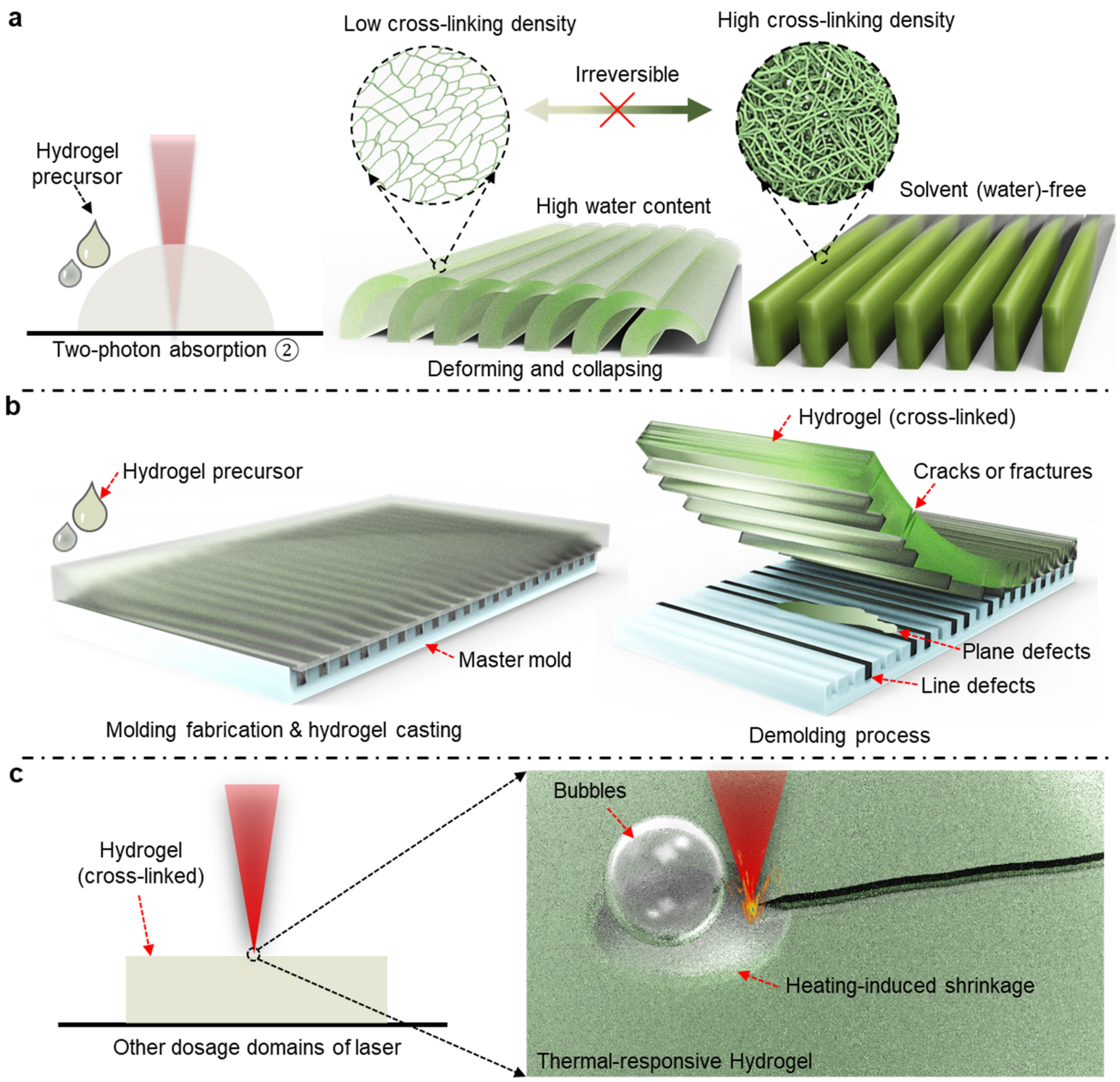

Supplementary Fig. 1 Schematic illustration showing the challenge of high-precision fabrication for hydrogel materials. a, Schematics of two-photo polymerization of hydrogels, including high water content and water-free recipes. **b,** Schematics of the molding and demolding process of hydrogels for replicating intricate structures. **c,** Schematic of direct laser-ablated hydrogel.

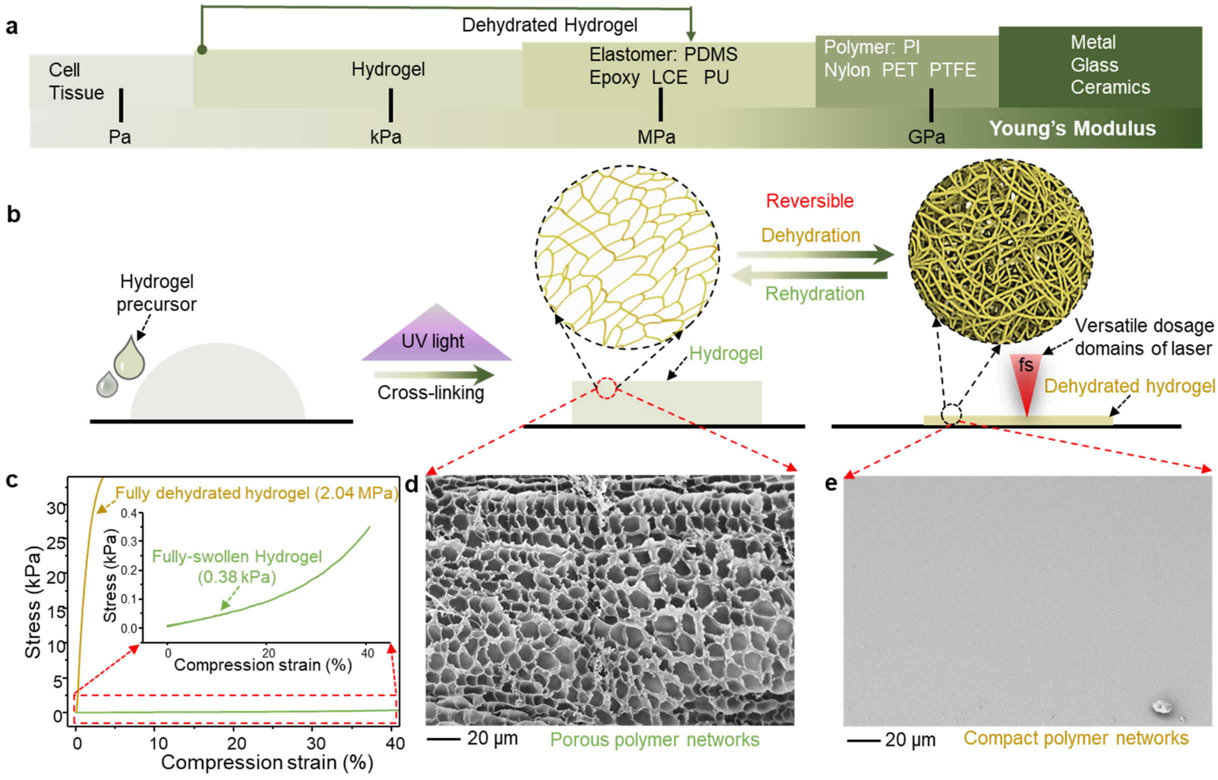

Supplementary Fig. 2 Dehydration of LIHAM Hydrogels enhances their structural integrity of the fabricated micro/nanostructures. a, Comparison of the mechanical properties of hydrogels with versatile common materials. **b**, Schematic illustration of micro/nanofabrication process of the LIHAM hydrogels. **c**, Compression test of the LIHAM hydrogel and the dehyrated hyrogel. **d**, Scanning electron microscopy (SEM) images of LIHAM hydrogels, showing the porous mophologies. **e**, SEM image of dehydrated LIHAM hydrogel, showing a smooth morphologies as a result of the closely compact polymer networks after dehydration.

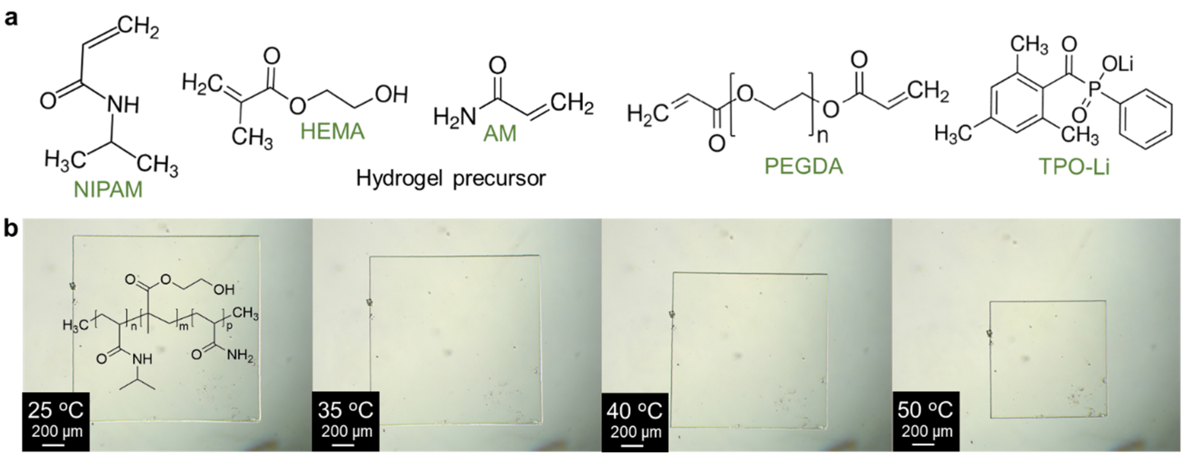

Supplementary Fig. 3 Thermally responsive LIHAM hydrogels. a, Main chemical components of the hydrogel precursor for making the LIHAM hydrogels. **b,** Optical images showing the deformation evolution of a detached hydrogel film in water. The hydrogel film remains transparent during the heating process.

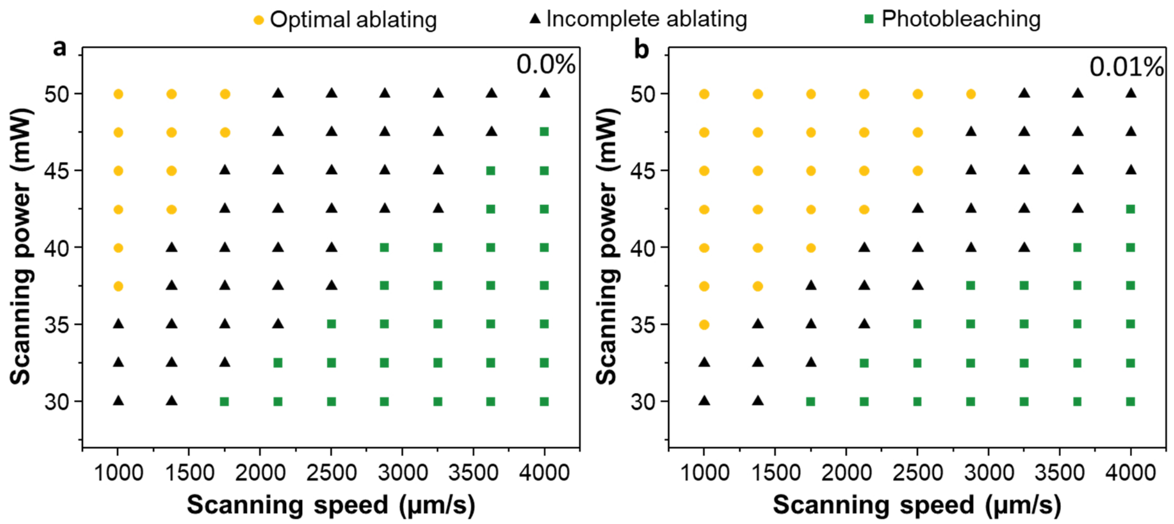

Supplementary Fig. 4 Effect of fluorescent molecule (phenol red) dopants on the written domains of the hydrogel film. a,b, Charts displaying three written domains (optimal ablating, incomplete ablating, and photobleaching domains) of a hydrogel film without (**a**) and with (**b**) fluorescent molecule dopant.

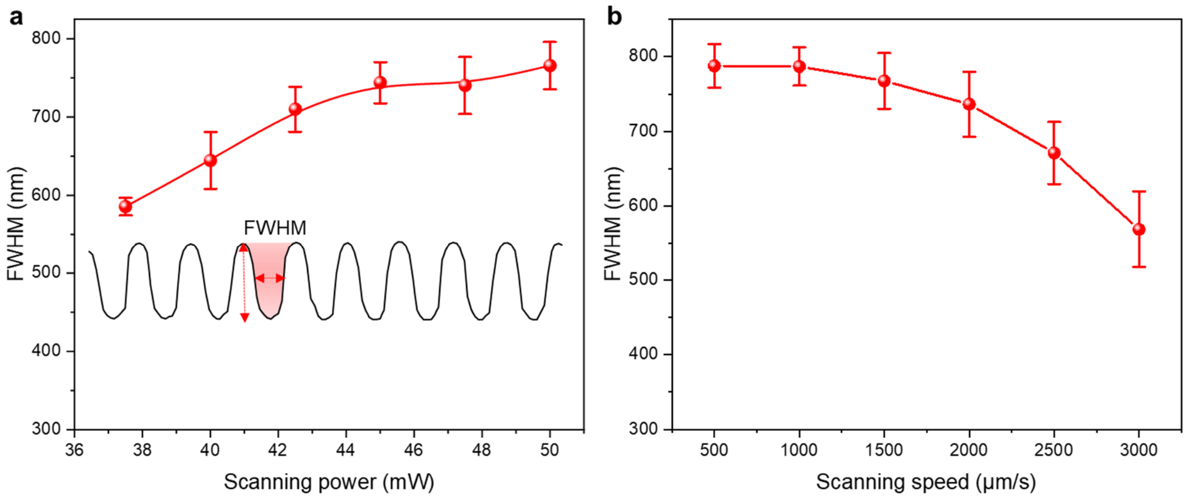

Supplementary Fig. 5 Effect of the scanning power (a) and speed (b) of the laser on the full width at half maximum (FWHM) of the obtained grooves. Data points are shown as mean ± s.d. (n=23 for **a**) and (n=18 for **b**).

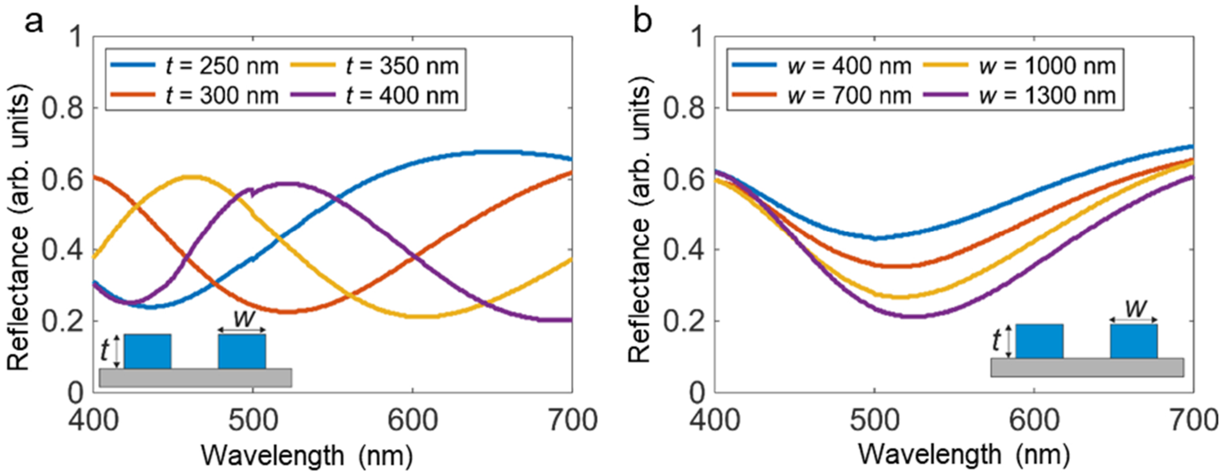

Supplementary Fig. 6 Calculated reflectance spectra depending on (a) the thickness and (b) the width of the proposed grating. The period of the grating is set to 1500 nm. The refractive index of substrate and the grating is 1.46 and 1.35, respectively.

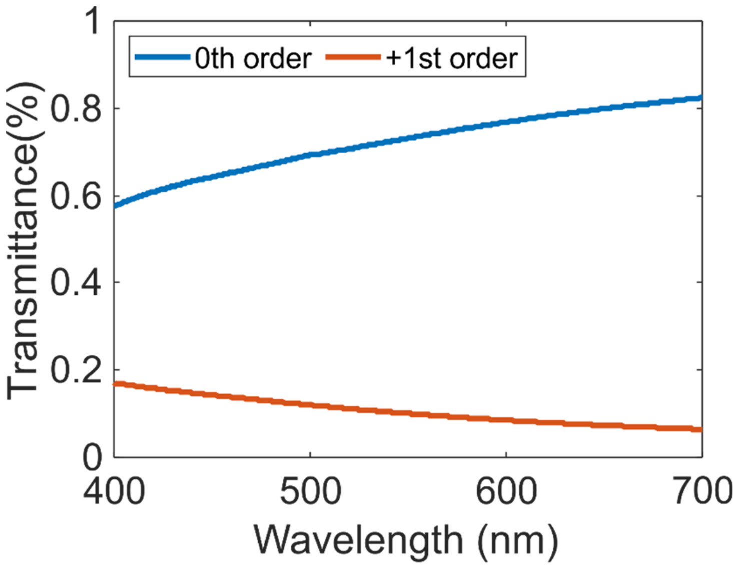

Supplementary Fig. 7 The calculated transmittance of the 0th order and +1st order diffraction by the proposed periodic gratings.

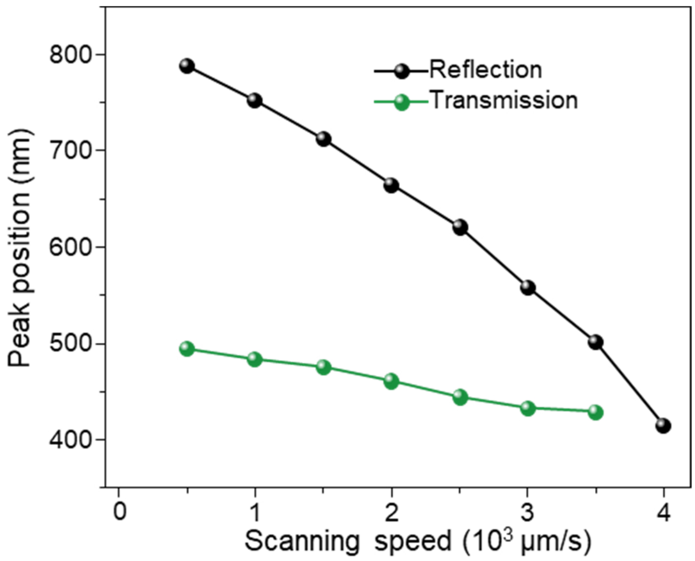

Supplementary Fig. 8 Effect of scanning speeds of the laser on the measured spectrum peaks of the corresponding grooves.

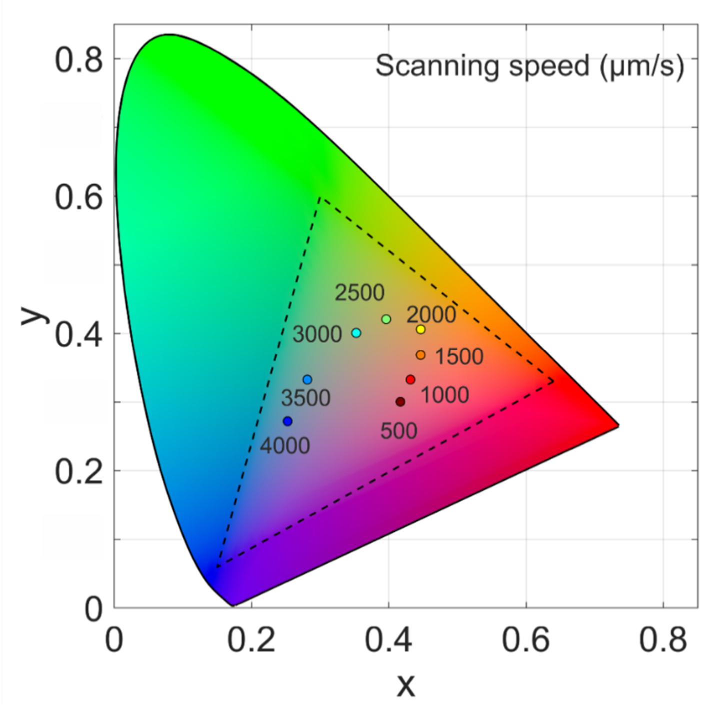

Supplementary Fig. 9 The calculated structural colors from the simulated reflection spectra of the structures with varying dimension in CIE 1931 chromaticity diagram.

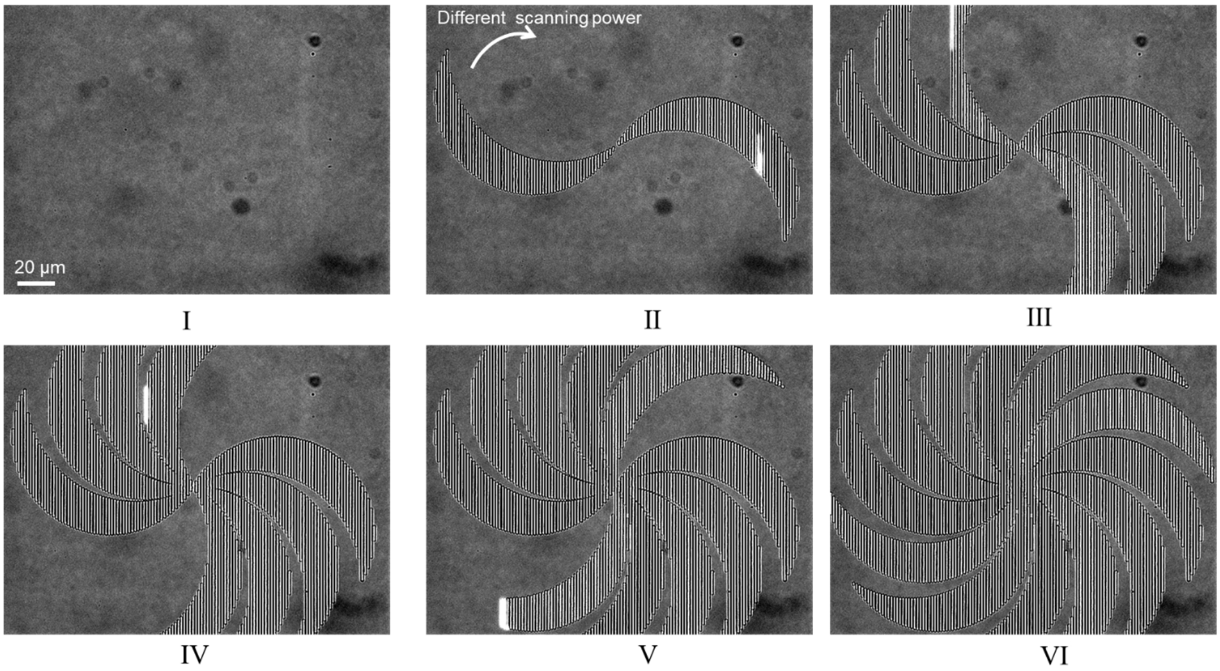

Supplementary Fig. 10 Fabrication process (I-VI) of a windmill pattern with different scanning powers in each section.

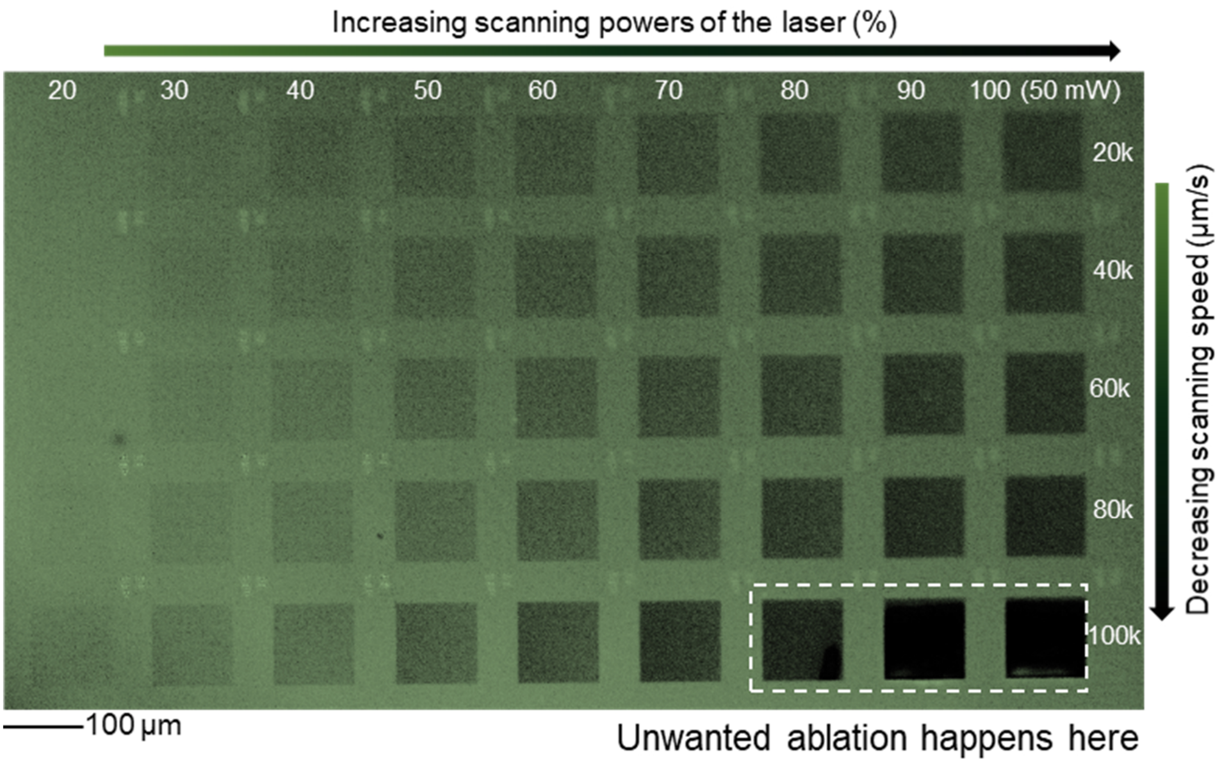

Supplementary Fig. 11 Fluorescent microscopy image displaying written squares with different scanning powers and scanning speeds.

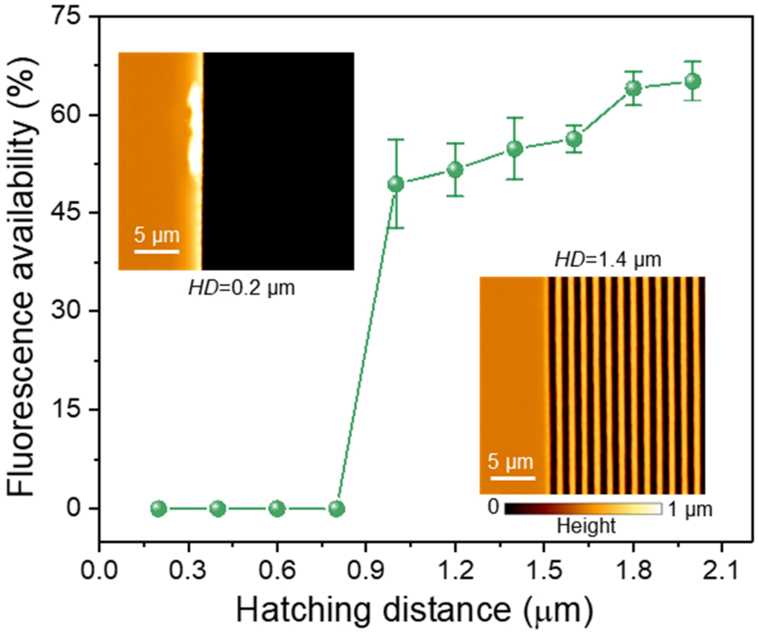

Supplementary Fig. 12 Effect of Hatching distance (*HD*) of written grooves on the fluorescence availability, where fluorescence availability is defined as the ratio of areas without ablation to the original whole areas. The insets are two typical atomic force microscopy images showing the resulted morphologies with two different *HD*s. Data points are shown as mean ± s.d. (n=14).

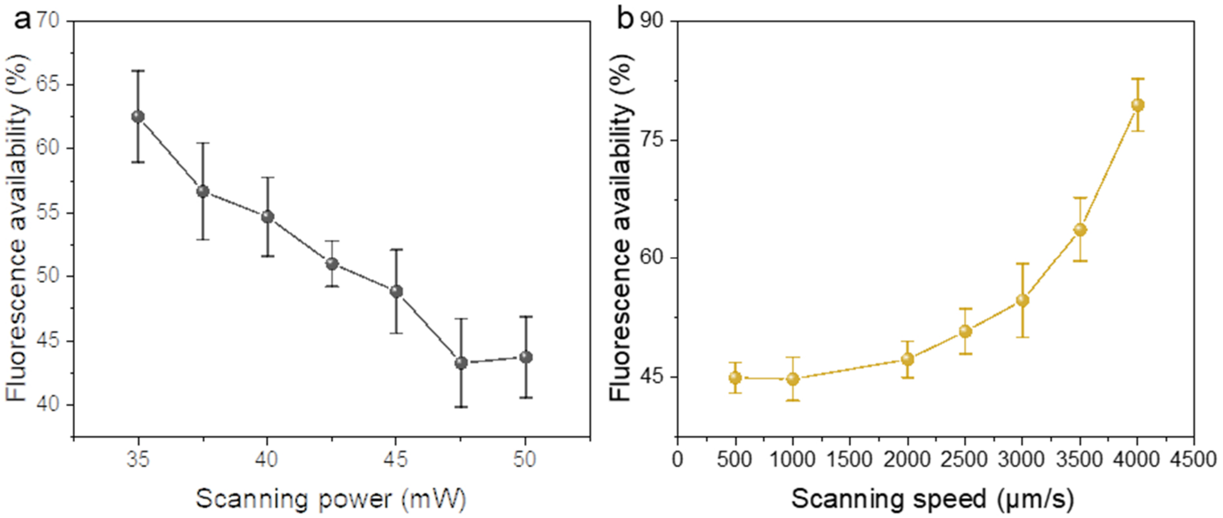

Supplementary Fig. 13 Effect of scanning powers (a) and speeds (b) of the laser for the groove ablation on the fluorescence availability. Data points are shown as mean ± s.d. (n=14 for **a** and **b**).

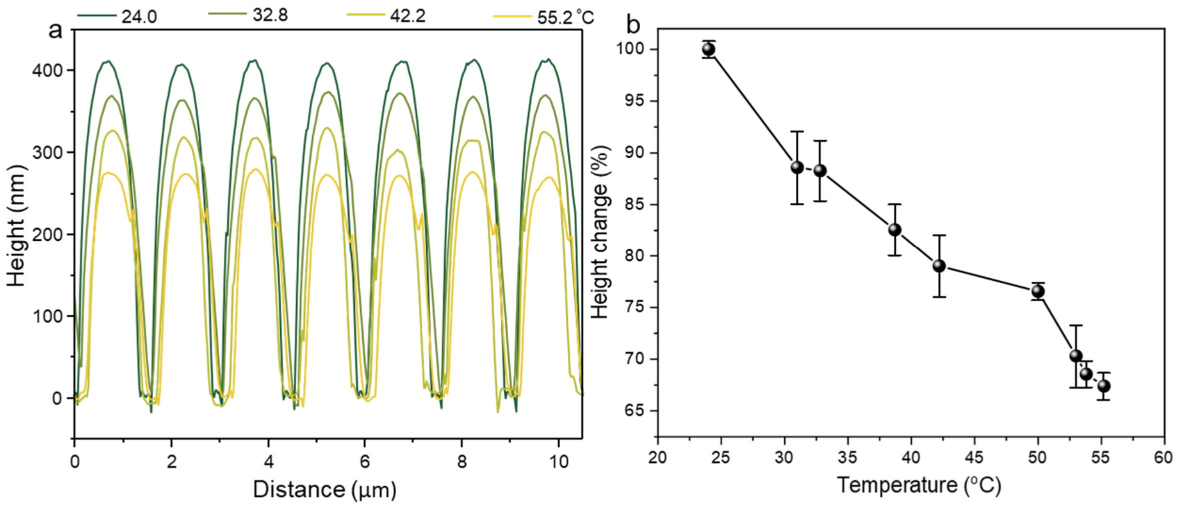

Supplementary Fig. 14 Temperature-responsiveness of the ablated grooves in water. a, Geometries of the ablated grooves measured by in-situ AFM at different temperatures. **b**, Changes in height of the grooves as the temperatures rise. Data points are shown as mean ± s.d. (n=11).

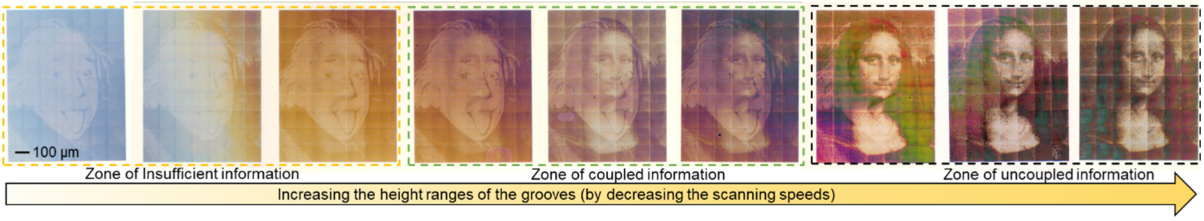

Supplementary Fig. 15 Reflective optical images showing the image decoupling of different channels (groove height and angle) through applying different scanning speeds. The Image information of *Mona Lisa* is encoded in the heights of grooves, and that of *Albert Einstein* is encoded in the angles of grooves. The image of *Albert Einstein* vanishes in reflective optical images only when certain height range is reached (but it could be observed under the cross-polarized microscope).

Supplementary References

1 He, S., Yu, J. & Hu, M. Femtosecond laser high precision fabrication for novel applications. *Curr. Nanosci.* **12**, 676-684 (2016).

2 Song, J., Michas, C., Chen, C. S., White, A. E. & Grinstaff, M. W. From simple to architecturally complex hydrogel scaffolds for cell and tissue engineering applications: Opportunities presented by two-photon polymerization. *Adv. Healthc. Mater.* **9**, 1901217 (2020).

3 Torgersen, J. *et al.* Hydrogels for two-photon polymerization: a toolbox for mimicking the extracellular matrix. *Adv. Funct. Mater.* **23**, 4542-4554 (2013).

4 Brigo, L. *et al.* 3D high-resolution two-photon crosslinked hydrogel structures for biological studies. *Acta Biomater.* **55**, 373-384 (2017).

5 Ceylan, H., Yasa, I. C. & Sitti, M. 3D Chemical Patterning of Micromaterials for Encoded Functionality. *Adv. Mater.* **29**, 1605072 (2016).

6 Ruan, Q. *et al.* Reconfiguring colors of single relief structures by directional stretching. *Adv. Mater.* **34**, 2108128 (2022).

7 Zhang, M. *et al.* Hydrogel muscles powering reconfigurable micro-metastructures with wide-spectrum programmability. *Nat. Mater.* **22**, 1243-1252 (2023).
