# Supplementary material for: Micro- and nanofabrication of dynamic hydrogels with multichannel information
Source: Nat Commun. 2023 Dec 11;14:8208. doi: 10.1038/s41467-023-43921-9 (PMC10713606; doi:10.1038/s41467-023-43921-9)
Supplement: Supplementary file 3 — Description of Additional Supplementary Files [file 41467_2023_43921_MOESM3_ESM.pdf]

Description of Additional Supplementary Files

File Name: Supplementary Movie 1
Description: Dual-information displaying of a written pattern on the hydrogel surface recorded under the cross-polarizers.

File Name: Supplementary Movie 2
Description: Sequential image displaying from Mona Lisa to Albert Einstein along the rise of temperature from 25 to 60°C in an aqueous environment.
