# Supplementary material for: Micro- and nanofabrication of dynamic hydrogels with multichannel information
Source: Nat Commun. 2023 Dec 11;14:8208. doi: 10.1038/s41467-023-43921-9 (PMC10713606; doi:10.1038/s41467-023-43921-9)
Supplement: Supplementary file 2 — Peer Review File [file 41467_2023_43921_MOESM2_ESM.pdf]

nature portfolio

Peer Review File

Micro- and nanofabrication of dynamic hydrogels with multi-channel information

Open Access This file is licensed under a Creative Commons Attribution 4.0 International License, which permits use, sharing, adaptation, distribution and reproduction in any medium or format, as long as you give appropriate credit to the original author(s) and the source, provide a link to the Creative Commons license, and indicate if changes were made. In the cases where the authors are anonymous, such as is the case for the reports of anonymous peer reviewers, author attribution should be to 'Anonymous Referee' followed by a clear attribution to the source work. The images or other third party material in this file are included in the article's Creative Commons license, unless indicated otherwise in a credit line to the material. If material is not included in the article's Creative Commons license and your intended use is not permitted by statutory regulation or exceeds the permitted use, you will need to obtain permission directly from the copyright holder. To view a copy of this license, visit http://creativecommons.org/licenses/by/4.0/.

REVIEWER COMMENTS

Reviewer #1 (Remarks to the Author):

In this manuscript, the authors use femtosecond laser pulses to encode different layers of information in a hydrogel. They demonstrate the individual effects of nanostructuring by ablation and photobleaching (using a different laser). As a result, information can be encoded in the independent "channels" of color, polarization state (affected by the ablated pattern), and fluorescence intensity (affected by bleaching).

The authors motivate the study by making use of dosage domains of fs laser irradiation that are conventionally not made use of in photoinduced reactions requiring two-photon absorption.
This is a reasonable motivation, but it raises questions about the scope and relevance of the research. These questions should be addressed before deciding whether it is suitable for the journal of interest.
- The individual effects of ablation, nanostructuring, and photobleaching are well known, and the material is not the main novelty of the paper either. Could the authors clarify the scientific advance beyond the superposition of well-known processes?

- Is there truly a point in using fs laser technology for the effects reported in the manuscript? I'd ask the authors to explain why complex fs laser technology should be used for printing structures and encoding directional information. A comparable resolution is achievable by much simpler means such as CO_2 lasers or excimer lasers. If (as stated in the motivation) two-photon processes are not required, the technological relevance of the approach based on fs lasers is questionable.

- Are the authors aiming for technological or scientific impact with this manuscript? How does it connect to the examples of micro/nano-fabrications mentioned above?

The underlying methodology is sound and the results are convincing. The data are sufficient to support the main conclusion that the three methods/channels can be combined. Besides that, the manuscript primarily provides values such as threshold intensities. For readers to be able to reproduce the results, the authors should provide more details about the laser beams (size, intensity distribution). This would also provide a better estimate of the total dose per spot.

Certain statements should be backed up better by data/literature or toned down:
- the claim that the thermal response of LIHAM provides the opportunity for dynamical tuning of structures, "providing a stimuli responsive means of manipulating the encoded information" is not backed up by the information provided.
- different images at the same location "providing unprecedented opportunities" also seems to be too strong a claim, with reported examples that even encode 3D structures (e.g.: Light: Science & Applications 11, 302 (2022)).

Additional remarks:
- Certain sections should be backed up by references, for example the photobleaching of dyes.
- It would be good to clarify what is the key parameter for each of the effects: dose, fluence, flux, or others.
- Could the authors provide more information on the mechanism of the induced changes (hot electrons vs. heat)?

Reviewer #2 (Remarks to the Author):

In this paper, the authors have detailed their work on multi-information writing in a hydrogel, LIHAMS, utilizing wavelength-dependent diffraction of ablated grooves, its polarization sensitivity, and photobleaching of fluorescent dyes. I am impressed by the careful optimization of laser irradiation conditions for each optical mode to inscribe information in xy dimensions, culminating in the successful recording of different information on a plate. The authors have adeptly integrated well-established principles of recording, physics, and photochemistry behind the technology with the material they developed. The manuscript is well articulated, and the technical demonstrations appear sound. Moreover, the experiments seem well-executed, as I could not identify any flaws in the procedures.

However, while the authors have skillfully brought together existing knowledge and their own material development, it is challenging to discern novel scientific findings in the current manuscript. It would enhance the paper significantly if the authors could elucidate what made this achievement possible, perhaps from a materials perspective, and highlight any novel scientific findings. Additionally, strengthening the introduction to appeal to a broader audience, as targeted by this journal, would be beneficial. With these improvements, I can recommend the publication of this paper in Nature Communications.

To facilitate better understanding of the research for readers, I suggest the inclusion of the following points:

- Detailed exposition of exposure conditions (intensity, scanning speed, lens specifications for writing and reading, etc.) for each experimental demonstration.
- A discussion on the capability of recording information in 3D space (x,y,z) using all modalities together.

I believe that addressing these points will provide a more comprehensive perspective of the research and render the manuscript more impactful for the broad readership of Nature Communications.

Reviewer #3 (Remarks to the Author):

This manuscript describes a laser processing method that combines three laser 2D patterning techniques to write different micro-structures on hydrogel surface, and to produce images based on structure color, polarization state, and fluorescent intensity. Visually impressive images were produced.
However, this work is a combination of three well, long-established methods. It lacks the novelty or innovation in terms of advancing the laser processing method (or enhancing the fundamental understanding of laser processes). Hence it is not suitable for publication in Nature Comm. Laser micromachining of sub-micrometer structures, grating like structures (including the laser-induced periodic surface structures or LIPSS) for displaying different colors, polarization dependent microstructures, and direct laser write of fluorescent microstructures all have been investigated and well-established for tens of years. (Also see below for the specific comments.) Additionally, the application of this work seems to be limited. In addition to those loosely mentioned at the beginning of the abstract, the only other relevant use has to do with the specific material in the author's group in Line 86-88. Even there, it is not clear how the fabricated structures will benefit LIHAMS. Therefore, the impact of this work is limited.
Here are some additional comments:
1. Title: "all-in-one" needs to be changed. There are other features or structures that this method does not create, such as true 3D structures (also see below).
2. Abstract and introduction part of the main text: The topic of this manuscript is very much different from two-photon printing, as it does not deal with true 3D printing at all. Comparison to 3D printing should be kept to a minimum, while many similar printing/laser processing methods (fabrication of microscale or sub-microscale grooves, fabricating of grating like periodic structures including LIPSS, etc.) should be discussed and referenced in abstract, intro, and other places. For example, Line 36, 3D data storage using TPP or direct laser writing was presented as early as 1989. Encryption using DLW has also been extensively reported as a plausible application for a long time. Combination of subtraction and addition has also been studied extensively. Literature on these topics should be reviewed and included, in reference to the novelty of the current work.
3. Line 21 should be "within the rest of the unablated areas"
4. Line 22 should be "three-channels of information" or "three-channel information"
5. Line 52, 243, 364, etc., the word "Besides" is used incorrectly and should be replaced with something like "Additionally".
6. The section starting from Line 67: in the description of the all-in-one lithography process, it seems the two-photon absorption region, or region 2 in Figure 1A is left unused. This should be addressed since discussion elsewhere in the text suggests region 2 is useful to this process.
7. Furthermore, line 109 suggests the two-photon absorption region should be avoided all together. This again shows it is unnecessary to provide extensive discussions in abstract and introduction of two-photon processes. Also, it would be better to clarify in the introduction, ~line 43, that the typical two-photon absorption regimes for cross-linking based additive manufacturing are avoided and only multi-photon ablation regimes for subtractive manufacturing are used.
8. Line 121, a minimum hatching distance of 1 um is relatively large compared to other lithography or laser direct write methods. Is this limitation attributed solely to the thermal dynamics of the hydrogel? If so, do the benefits of the thermal sensitivity of the LIHAM hydrogel offset the disadvantage of limiting the hatching distance?

9. Line 220, why does larger HD increase dosage of the laser? Are the authors implying these two are disconnected and larger HD requires more dosage be used? Otherwise, larger HD should decrease the dosage of the laser in a given volume when all else is constant.
10. Figure 5E shows fluorescent retention increases as HD increase not the other way around as was stated in the text, Line 221.
11. Line 277, dynamic switching between the image channels implies reversibility. Can all three channels of information be reobtained after the LIHAM hydrogel is heated/cooled? Is the Mona Lisa information in Figure 6K kept or lost after the heating is completed?
12. Outlook, Line 296: again, if this process is to be compared directly to 2PP the 2D and fully-subtractive aspect of this work must be acknowledged. Again, a significant advantage of 2PP is the ability to additively create true 3D sub-micro structures. This work only presents 2D structures.
13. Line 371, the impressive capabilities of the Nanoscribe were utilized, but many specifics of the Nanoscribe parameters are available to public. More details on the experimental setup should be provided so that this work can be repeated on any DLW experimental setup, i.e., laser specifications, optical setup (objective lens, dip-in or oil-immersion printing, and laser scanning hardware etc.

Response to Reviewers' comments

We express our gratitude to the reviewers for providing us with their insightful and constructive feedback on our manuscript. We have modified and revised our manuscript based on the reviewers' comments and suggestions, and hope to have adequately addressed all the questions and concerns raised by the reviewers. We believe that these revisions have substantially improved the clarity of our work and highlighted its scientific contributions. To facilitate the reviewers' access to our responses, we have provided point-by-point replies to their comments, which are indicated by blue arrows (→) below. Additionally, we have listed all changes made in either the main text or Supplementary Information files of our manuscript and highlighted them in yellow.

Reviewer #1:

Summary

In this manuscript, the authors use femtosecond laser pulses to encode different layers of information in a hydrogel. They demonstrate the individual effects of nanostructuring by ablation and photobleaching (using a different laser). As a result, information can be encoded in the independent "channels" of color, polarization state (affected by the ablated pattern), and fluorescence intensity (affected by bleaching). The authors motivate the study by making use of dosage domains of fs laser irradiation that are conventionally not made use of in photoinduced reactions requiring two-photon absorption. This is a reasonable motivation, but it raises questions about the scope and relevance of the research. These questions should be addressed before deciding whether it is suitable for the journal of interest.

→We appreciate the reviewer's accurate summary and kind suggestions. We have thoroughly revised our manuscript according to the reviewer's constructive suggestions, and believe its quality has been significantly improved.

Comment #1:

The individual effects of ablation, nanostructuring, and photobleaching are well known, and the material is not the main novelty of the paper either. Could the authors clarify the scientific advance beyond the superposition of well-known processes?

→We thank the reviewer for raising the concern. In our initial manuscript, we have given unnecessary discussion about two-photon polymerization, and we failed to present the main contribution and novelty of

this work. In response to the insightful comments from the reviewer, we conducted additional experiments and analyses to further underscore the novelty and significance of our study. To better clarify the contribution and further strength the innovation of our work, we have summarized the innovative aspects of our work into the following **key points (1-3)**:

(1) Our work introduces **a general strategy for the micro/nanofabrication of hydrogels**. Noted by their high content of water (typically exceeding 90% by weight), hydrogels are recognized for **their inherent softness**. While the softness of hydrogels offers unique advantages, such as tissue-compatibility, it also poses **significant challenges when it comes to creating micro/nanostructures within them.** Micro/nanostructures produced within hydrogels using various fs laser-based techniques, such as two-photon polymerization of hydrogel precursors, hydrogel molding, and direct laser ablation, often **lack the sufficient self-support, leading to structural instability and collapse.** Additionally, the **thermal effects** induced by fs lasers inevitably result in **undesirable deformations in hydrogels**, stemming from either the hydrogels' thermoresponsive properties or the laser heating-induced bubbles, thereby introducing an additional layer of **complexity to the precise fabrication** of hydrogel structures. In response, we have proposed a strategy by **dehydrating hydrogels**, enabling **high-precision manufacturing of hydrogel materials**. Dehydration not only **reinforces the self-support** of fabricated structures (the modulus enhances more than three orders of magnitude), but also temporarily **arrest or "freeze" their dynamic behaviors** during the laser fabrication process. To illustrate the efficacy of our strategy, we have applied it to a recently developed thermoresponsive hydrogel by our group, successfully achieving the **encoding of multi-information within the hydrogels.**

(2) Our approach yields microstructures with the remarkable capability **to simultaneously display multiple independent sets of information at the same location within hydrogels**, achieved through the use of **versatile dosage domains of fs laser**. While previous research has individually explored fs-laser micromachining to create intricate structures capable of exhibiting various characteristics such as different colors, polarization-dependent microstructures, and direct laser writing of fluorescent patterns, **the integration of these multi-channel information displays** at a single location using versatile laser dosage **has been a rare occurrence**. Through systematic design and optimization for each distinct channel within the same location, achieved by rationally applying different fs laser dosages, we have successfully achieved their **independent displays with minimal cross-talk effects**.

(3) Our thermoresponsive hydrogel, embedded with encoded multi-information, offers **a means of stimuli-responsive manipulation of the encoded information**. The dehydrated hydrogels can be restored to their original state (a water content exceeding 90%) through rehydrating inside water. As these compact

hydrophilic polymer networks regain their original porous network structure upon absorbing water, the **thermoresponsive behavior recovers**. Given that the multi-channel information is sensitive to the geometric parameters, such as the height of the as-fabricated structures, **thermal alterations** in these geometries enable **dynamic changes in the displayed information**. For instance, raising the temperature of the hydrogel **sequentially displays different images across different encoded channels**.

Consequently, we have made substantial revisions to our original manuscript, emphasizing these key novelty points. **We have also conducted new experiments, redesigned our main figures, and rewritten the introduction section of the manuscript.** Through this clarification of the novelties, we believe that our work represents a meaningful contribution to the field.

In the main text:

(Page 1, Line 13) Creating micro/nanostructures containing multi-channel information within responsive hydrogels presents exciting opportunities for dynamically changing functionalities. However, fabricating these structures is immensely challenging due to the soft and dynamic nature of hydrogels, often resulting in unintended structural deformations or destruction. Here, we demonstrate that dehydrated hydrogels, treated by a programmable femtosecond laser, can allow for a robust fabrication of micro/nanostructures. The dehydration enhances rigidity of the hydrogels and temporarily locks the dynamic behaviors, significantly promoting their structural integrity during the fabrication process.

(Page 2, Line 32) Micro/nanofabrication techniques have garnered significant attention across various fields, including integrated electronics, optics[1,2], miniature robotics[3-6], and microelectromechanical systems (MEMS)[7]. Among the tools employed for fabricating tiny structures[8,9], femtosecond (fs) lasers stand out as highly efficient, versatile and extensively utilized strategies[10,11]. The distinguishing feature of fs lasers lies in their ability to deliver short pulse durations with exceptionally high peak intensity[12,13]. This characteristic facilitates precise energy deposition while minimizing heat transfer to the surrounding areas[14]. As a result, fs lasers empower the creation of high-precision structures across a wide spectrum of materials[11]. The versatility inherent in the photon-matter interactions achievable with fs lasers confers distinct advantages in micro/nanofabrication[12,15-17]. Processes such as photobleaching[17], two-photon polymerization[11], and multi-photon ablation[13] enable tailored material modifications, substantially expanding the fabrication toolbox. Therefore, exploration and utilization of the diverse dosage domains offered by fs lasers opens up new possibilities in fabrication, presenting exciting opportunities for the integration of miniature devices across many applications.

However, despite the versatility of fs laser-based fabrication processes across a diverse range of materials, the creation of hydrogel micro/nanostructures poses a challenge[18,19]. Hydrogels, composed of three-dimensional (3D) hydrophilic polymer networks containing a high-water content (typically more than 90% by weight), have gained widespread attention owing to their versatile and easily tunable properties[20-22]. Nonetheless, the inherent softness of hydrogel materials, often characterized by low stiffness (typically on the order of kPa), presents significant hurdles in their high-precision fabrication[23,24]. The micro/nanostructures created within hydrogels often lack sufficient self-support, leading to structural instability and collapse[25]. Moreover, the inevitably generated thermal effects induced by the laser contribute to unwanted deformations of hydrogels, which arise either from the hydrogels' thermoresponsive properties or the laser heating-induced bubbles, adding another layer of complexity to the precise fabrication of hydrogel structures[26,27]. These challenges compromise structural integrity, imposing formidable obstacles in achieving high-precision structures within hydrogels, restricting the potential applications of miniature devices fabricated from hydrogels.

Here, we introduce a general strategy that allows for the micro/nanofabrication within soft and dynamic hydrogels by dehydrating the hydrogel. The dehydration of the hydrogel does not only bolster self-support of the fabricated structures but also transiently freezes their dynamic behaviors during the fabrication process. The versatility offered by various dosage domains of a fs laser empowers the creation of structures that can concurrently encompass multi-channel information within the responsive hydrogel, and exhibit multiple independent information at the same location on the hydrogel. Furthermore, we illustrate the dynamic capability of these structures in displaying intricate images sequentially through thermal activation of the hydrogel.

(Page 3, Line 72) **Micro/nanofabrication of multi-information within active hydrogels**

While the versatility offered by fs lasers in enabling different photon-material interactions (**Fig. 1a**), the fabrication of intricate structures in soft hydrogels remains a formidable challenge. Hydrogel materials are known for their inherent softness (on the order of kPa). While the softness of hydrogels offers unique advantages, it also presents significant hurdles when it comes to creating micro/nanostructures. Numerous strategies have been attempted for micro/nanofabrication of hydrogels using fs lasers, including two-photon polymerization of hydrogel precursors (Fig. 1b and Supplementary Fig. 1a), hydrogel molding (Supplementary Fig. 1b), and direct laser ablation of hydrogels (Supplementary Fig. 1c). Unfortunately, these approaches have fallen short in achieving high-precision micro/nanostructures with high structural integrity in hydrogels (see detailed discussion in Supplementary Note 1). We proposed a method to bolster their mechanical rigidity that relies on a reversible dehydration process of hydrogels (Fig. 1c and Supplementary

Fig. 2). This process results in the compaction of the original porous network within the hydrogel, rendering a substantial enhancement in mechanical properties. Furthermore, the dynamic behavior of hydrogels can be temporarily arrested or "frozen", contributing to an augmented structural integrity during the fs laser fabrication process. These dehydrated hydrogels with intricately fabricated structures by fs laser, can recover their original porous network (high water content) and stimuli-response through rehydrating in water.

(Page 10, Line 290) While the softness of hydrogels offers distinct advantages, it also poses significant challenges when it comes to creating micro/nanostructures. In our work, a general strategy of hydrogel dehydration has been implemented before fs laser fabrication, which significantly increases the structural integrity of the resulting micro/nanostructures.

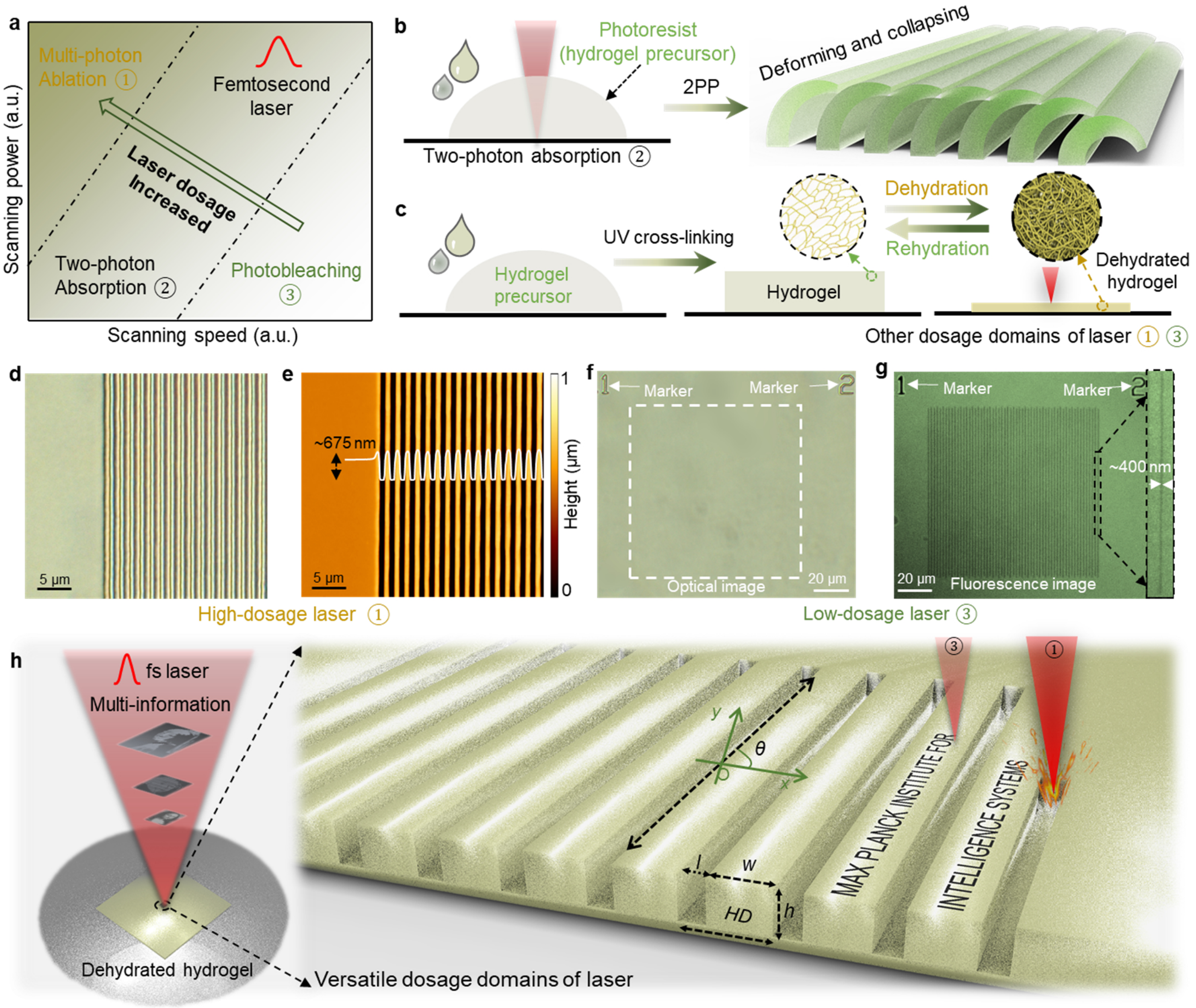

(Page 16, Line 483) **Fig. 1 Dehydration of thermoresponsive hydrogels enabling their high-precision fabrication for encoding multi-information using multi-dosage of the femtosecond (fs) laser. a**, Versatile dosage domains of the fs laser and their different uses in the photon-material interactions. **b**, Schematics showing the two-photon polymerized hydrogels are prone to deform or collapse due to the low

rigidity of hydrogels. **c**, Schematic showing the dehydrated hydrogels enhance the structural integrity during their fs laser fabrication. **d**, Optical image depicting the grooves on a hydrogel surface fabricated using laser with a high-dosage domain. **e**, Atomic force microscopy (AFM) image showing these written grooves on the hydrogel surface. **f**, Optical images illustrating a hydrogel surface using a low-dosage domain. The numbers "*1*" and "*2*" are abated as markers. **g**, Confocal fluorescence microscopy image with hydrogel surface treated in the low-dosage domain. **h**, Schematics of the micro/nanofabrication of the dehydrated hydrogel film with encoded with multi-information.

In the Supplementary Information:

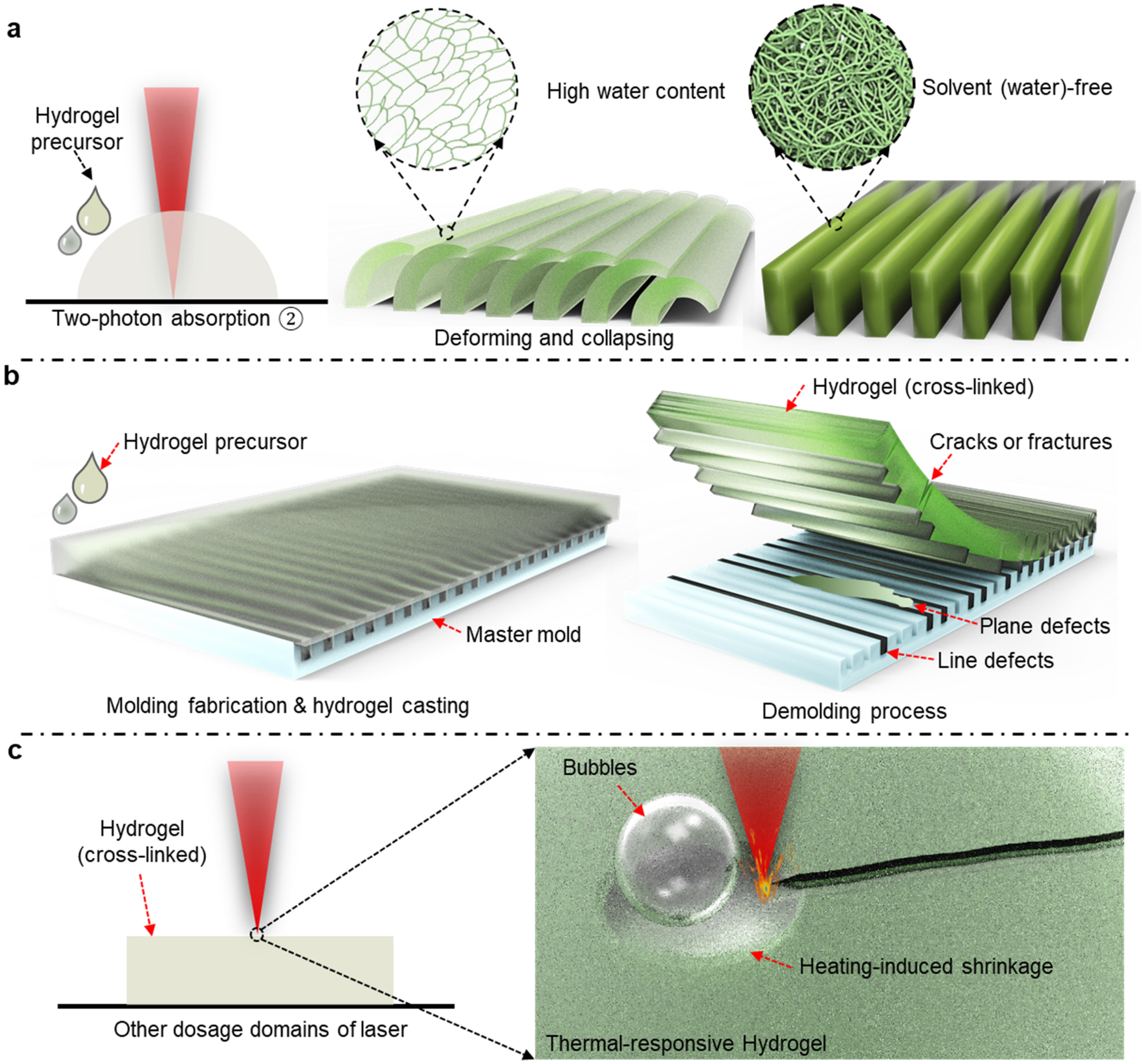

(Page 3, Line 79) **Supplementary Fig. 1 Schematic illustration showing the challenge of high-precision fabrication for hydrogel materials. a**, Schematics of two-photo polymerization of hydrogels, including high

water content and water-free recipes. **b,** Schematics of the molding and demolding process of hydrogels for replicating intricate structures. **c,** Schematic of direct laser-ablated hydrogel.

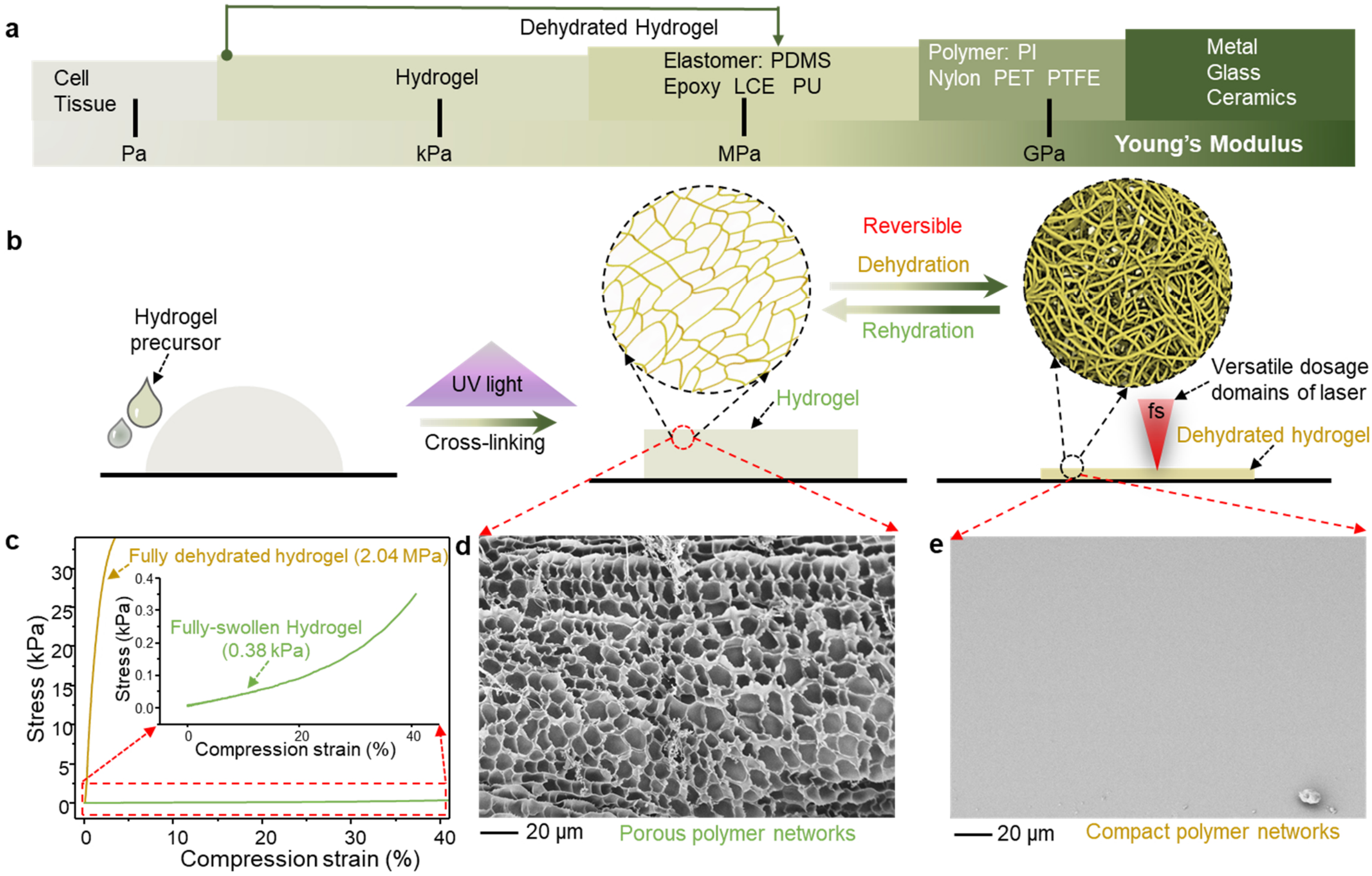

(Page 5, Line 86) **Supplementary Fig. 2 Dehydration of LIHAM Hydrogels enhances their structural integrity of the fabricated micro/nanostructures. a**, Comparison of the mechanical properties of hydrogels with versatile common materials. **b**, Schematic illustration of micro/nanofabrication process of the LIHAM hydrogels. **c**, Compression test of the LIHAM hydrogel and the dehyrated hyrogel. **d**, Scanning electron microscopy (SEM) images of LIHAM hydrogels, showing the porous mophologies. **e**, SEM image of dehydrated LIHAM hydrogel, showing a smooth morphologies as a result of the closely compact polymer networks after dehydration.

(Page 2, Line 19) **Supplementary Note 1. Typical stragies using femtosecond laser for the micro- and nanofabrication of hydrogel materials**

Femtosecond (fs) lasers have emerged as a highly efficient and versatile strategy for fabricating micro- and nanostructures across a wide range of materials[1]. This technique leverages versatile photon-matter interactions and encompasses both additive and subtractive manufacturing approaches.

Additive Manufacturing: One notable additive manufacturing approach for creating high-precision hydrogels is known as two-photon polymerization (2PP)[2,3]. In this process, a hydrogel precursor containing dispersed monomers in a solvent, typically water, undergoes site-specific crosslinking through two-photon absorption induced by the fs laser. This results in the creation of high-precision 3D structures at submicron scales (see Supplementary Fig. 1a). However, hydrogel structures produced through 2PP, especially those with high aspect ratios, are susceptible to deformation or collapse due to their low stiffness[4]. This is primarily a consequence of their high water content (exceeding 90%). While solvent-free hydrogel precursors can improve structural integrity due to their stiff 3D polymer structures[5], this also leads to much higher cross-linking density of networks. As a consequence, the later water absorption is limited due to the high cross-linking density, resulting in a departure from typical hydrogel properties like softness and responsiveness based on water adsorption/desorption, which are permanently compromised.

Another additive manufacturing strategy involves molding precision structures from a 2PP-fabricated master mold[6]. Hydrogel precursor solution is cast onto the master mold, cross-linked with UV light, and then detached (Supplementary Fig. 1b). However, this method is also hampered by the softness of hydrogel materials, leading to problems like line/plane defects and cracks during demolding process due to the fragile and soft nature of hydrogels.

Subtractive Manufacturing: Subtractive manufacturing techniques involve directly exposing materials to fs laser ablation, allowing for the creation of high-precision structures. Nevertheless, the high water content and dynamic behavior of hydrogels pose significant challenges to achieving precision in this process. Laser-induced heating leads to intense water evaporation (generating disturbing bubbles) within the hydrogel polymer networks, causing deformation and defects during ablation. Additionally, certain hydrogels, such as thermally responsive variants, are sensitive to temperature changes, further undermining structural integrity.

Our Dehydration-Based Strategy: As structures made of most commercial photoresits are usually rigid (usually with moduli on the order of GPa), they can achieve high-precision farbication. Therefore, we believe that as materials become increasingly rigid, the fabricated high-precision structures tend to become more self-supporting, thereby enhancing the structural integrity of the resulting micro/nanostructures (Supplementary Fig. 2a). Therefore, it can be advantageous to improve their mechanical properties by dehydrating the hydrogels before laser processing, as illustrated in Supplementary Fig. 2b. The dehydration process serves a dual purpose: it not only enhances the mechanical properties (Young’s modulus) of hydrogels more than three orders of magnitude (Supplementary Fig. 2c), but also temporarily stabilizes their dynamic behavior. The reason behind this effect lies in the transformation of the porous 3D network of

hydrogels into a more compact structure after dehydration (Supplementary Figs. 2d and 2e). The stabilization is particularly beneficial for responsive hydrogels, such as the one we have recently developed (LIHAM)[7]. As the thermal response of these hydrogels is governed by the desorption/adsorption of water, the dehydration process (absence of water) can effectively arrest or freeze their thermal response.

Importantly, this dehydration does not alter the cross-linking density of the hydrogel. As a result, the dehydrated hydrogel can recover its high water content after micro/nanofabrication, thus retaining its thermal responsiveness. In this way, this reversible dehydration/rehydration process enables dynamic and reconfigurable functionalities in the fabricated structures. Our strategy offers a solution to the challenges associated with precision hydrogel manufacturing using fs lasers, opening up new possibilities for the dynamic and responsive design of hydrogel-based miniature structures and devices.

Comment #2:

Is there truly a point in using fs laser technology for the effects reported in the manuscript? I'd ask the authors to explain why complex fs laser technology should be used for printing structures and encoding directional information. A comparable resolution is achievable by much simpler means such as CO_2 lasers or excimer lasers. If (as stated in the motivation) two-photon processes are not required, the technological relevance of the approach based on fs lasers is questionable.

→We appreciate the reviewer's concern and would like to clarify the rationale behind our choice of femtosecond (fs) laser technology for our work. CO_2 lasers are not suitable as light sources to achieve the desired resolution in our research because in principle they have long wavelengths around 9.3-10.6 μm. This longer wavelength limits their ability to achieve very high resolution compared to shorter-wavelength lasers. Rather, CO_2 lasers are often used for cutting and engraving materials such as wood, acrylic, and various metals, where the resolution is not as critical as in some other applications. On the other hand, excimer lasers emit short UV wavelengths, typically ranging from 126 nm to 351 nm, depending on the specific type of excimer gas used. They are widely employed in semiconductor manufacturing for photolithography processes, where achieving high resolution is imperative for creating intricate patterns on silicon wafers. Excimer lasers are renowned for their capability to achieve extremely high resolutions in photolithography, often reaching resolutions on the order of tens of nanometers or even finer. However, realizing such high resolution demands precise control over laser beam characteristics, intricate optics, and the use of advanced photomasks. While excimer laser technology offers the potential for exceptional resolution, it's important to acknowledge that it can also incur elevated costs, longer manufacturing times, and may require specialized

programming and equipment due to the intricacies of achieving the desired results. Here, several factors why we use fs laser are listed below:

1. **High Precision**: fs lasers emit extremely short pulses in the femtosecond range, allowing for precise material ablation with minimal collateral damage. This level of precision is crucial for the creation of intricate micro/nanostructures and encoding directional information with high fidelity.

2. **Cold Ablation**: Fs lasers enable "**cold**" ablation, meaning that material removal occurs with minimal heat generation. This characteristic is particularly important when working with delicate or heat-sensitive materials, such as our thermal responsive and soft hydrogel materials, ensuring minimal thermal distortion or damage.

3. **Programmability and Versality**: Our research focuses on not only achieving high-resolution structures but also encoding multi-information within hydrogels. The high programmability fs laser integrated in the Nanoscribe system facilitates the creation of complex structures and the encoding of multi-information into multi-channels. Additionally, the versatility of fs lasers can be applied to a wide range of materials and applications, offering flexibility in our research efforts.

While we acknowledge that simpler laser technologies may suffice for some applications, our choice of fs laser technology aligns with our goal of achieving high resolution, versatility in the creation of micro/nanostructures and multi-information encoding. We believe that the unique capabilities of fs lasers have enabled us to achieve the sophisticated functionalities presented in our manuscript. Accordingly, we added these discussions in the revised manuscript.

In the main text:

(Page 2, Line 32) Micro/nanofabrication techniques have garnered significant attention across various fields, including integrated electronics, optics[1,2], miniature robotics[3-6], and microelectromechanical systems (MEMS)[7]. Among the tools employed for fabricating tiny structures[8,9], femtosecond (fs) lasers stand out as highly efficient, versatile and extensively utilized strategies[10,11]. The distinguishing feature of fs lasers lies in their ability to deliver short pulse durations with exceptionally high peak intensity[12,13]. This characteristic facilitates precise energy deposition while minimizing heat transfer to the surrounding areas[14]. As a result, fs lasers empower the creation of high-precision structures across a wide spectrum of materials[11]. The versatility inherent in the photon-matter interactions achievable with fs lasers confers distinct advantages in micro/nanofabrication[12,15-17]. Processes such as photobleaching[17], two-photon polymerization[11], and multi-photon ablation[13] enable tailored material modifications, substantially expanding the fabrication toolbox.

Therefore, exploration and utilization of the diverse dosage domains offered by fs lasers opens up new possibilities in fabrication, presenting exciting opportunities for the integration of miniature devices across many applications.

Comment #3:

Are the authors aiming for technological or scientific impact with this manuscript? How does it connect to the examples of micro/nano-fabrications mentioned above?

→We thank the reviewer for raising the concerning. We believe our work presents a general strategy for nano/microfabricating hydrogels. Hydrogels, known for their high water-content (typically exceeding 90% by weight), possess inherent softness. While this softness offers unique advantages such as tissue compatibility, it presents significant challenges when attempting to create micro/nanostructures within them. Various fs laser-based techniques, including two-photon polymerization of hydrogel precursors, hydrogel molding, and direct laser ablation, often result in insufficient self-support for the micro/nanostructures within hydrogels, leading to structural instability and collapse. Moreover, the thermal effects induced by fs lasers can lead to unwanted deformations in hydrogels, either due to the hydrogels' thermoresponsive properties or the formation of bubbles, adding complexity to precise hydrogel structure fabrication.

In response to these challenges, we have proposed a strategy involving the dehydration of hydrogels, enabling the high-precision manufacturing of hydrogel materials. Dehydration not only enhances the self-support of fabricated structures but also temporarily halts or "freezes" their dynamic behaviors during the laser fabrication process. To demonstrate the effectiveness of our approach, we applied it to a thermoresponsive hydrogel recently developed by our research group, successfully enabling the encoding of multiple types of information within the hydrogels. Therefore, we believe our strategy to enable the micro/nanofabrication of hydrogels is the basis for the following fs laser-processed versatile examples. This advancement holds significant promise for the field of fs laser manufacturing, particularly for soft materials like hydrogels. We have also conducted new experiments, redesigned our main figures, and rewritten the introduction section of the manuscript. For a more detailed examination of these revisions, please refer to our comprehensive response in the "**Reply to the Comment #1**" section.

Comment #4:

The underlying methodology is sound and the results are convincing. The data are sufficient to support the main conclusion that the three methods/channels can be combined. Besides that, the manuscript primarily

provides values such as threshold intensities. For readers to be able to reproduce the results, the authors should provide more details about the laser beams (size, intensity distribution). This would also provide a better estimate of the total dose per spot.

→We appreciate the reviewer's question and provide additional details about the laser and objective we used in our study. The pulsed laser we employed had the following parameters: a wavelength of 780 nm, pulse duration of 80 femtoseconds, and a repetition rate of 80 MHz. For printing the micro/nanostructures, we utilized a 63X immersion objective (NA=1.4, focal Width (FWD)=0.19 mm, parfocal Length=45.06 mm, Zeiss). These parameters can be used to estimate the beam size based on ideal Gaussian beam behavior. Additionally, we conducted measurements of the full width at half maximum (FWHM) of the ablated grooves (obtained by linearly scanning a single laser spot) at various scanning powers and speeds. These FWHM values of ablated grooves are the result of both the laser beam size and intensity. We have incorporated these details and relevant discussions into the revised manuscript.

In the main text:

(Page 5, Line 138) Modifying the dosage of laser by increasing the scanning power or reducing the scanning speed can tailor the morphologies of the obtained grooves and result in the creation of a deeper groove (Figs. 2e, f) and a broader full width at half maximum (FWHM) of the groove (Supplementary Fig. 5).

(Page13, Line 414) The dehydrated LIHAM film on the glass substrate was then directly written through the fs laser (center wavelength: 780 nm, pulse duration: 80 fs, repetition rate: 80 MHz, average output power (scanning power): 0-50 mW) with programmable dosage domains and writing processes (DeScribe software, Nanoscribe GmbH). We used a 63X oil-immersion objective (NA=1.4, focal width (FWD)=0.19 mm, parfocal length=45.06 mm, Zeiss) to enable the micro/nanofabrication within the LIHAM film.

In the Supplementary Information:

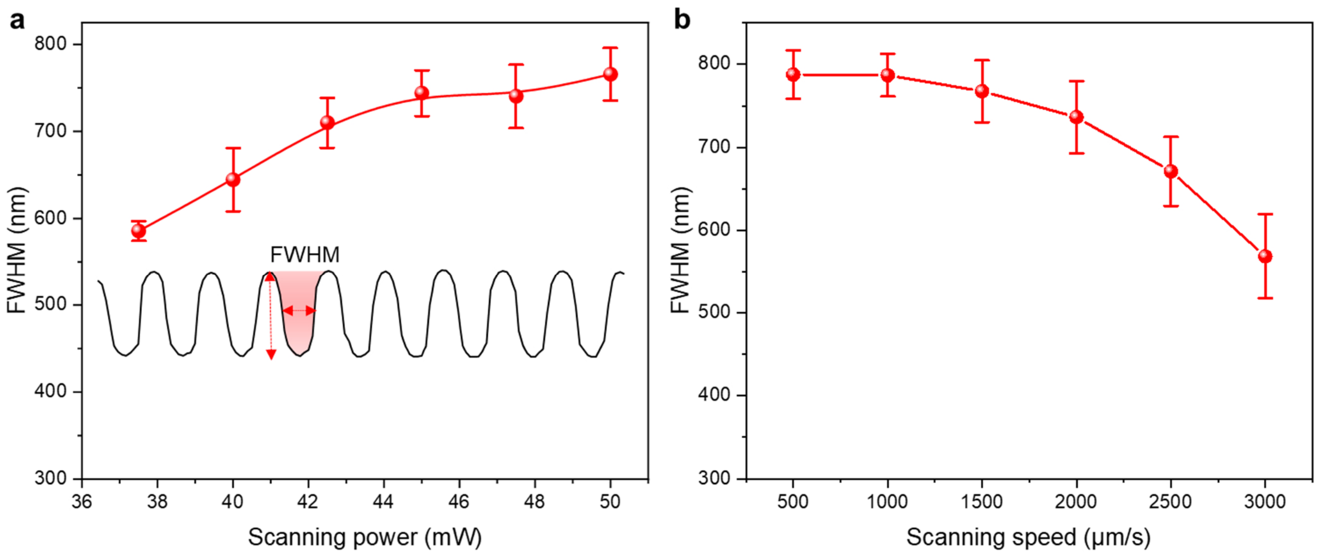

(Page 8, Line 108) **Supplementary Fig. 5 Effect of the scanning power (a) and speed (b) of the laser on the full width at half maximum (FWHM) of the obtained grooves.**

Comment #5:

Certain statements should be backed up better by data/literature or toned down:

A: the claim that the thermal response of LIHAM provides the opportunity for dynamical tuning of structures, "providing a stimuli responsive means of manipulating the encoded information" is not backed up by the information provided.

B: different images at the same location "providing unprecedented opportunities" also seems to be too strong a claim, with reported examples that even encode 3D structures (e.g.: Light: Science & Applications 11, 302 (2022)).

→We appreciate the reviewer for the constructive suggestions. For Comment #5-A, we have actually provided the data regarding the thermal response of LIHAM in Supplementary Information Fig. 14 (Atomic force microscopy data). Additionally, we demonstrated the capability to switch between different images as the temperature rises in Figs. 6k, l, and Supplementary Video 2. This dynamic behavior allows us to sequentially display two different images from image of *Mona Lisa* to that of *Albert Einstein*. Please refer to the relevant discussion starting from Page 9, Line 268, to Page 10, Line 292, for further details.

For Comment #5-B, we have made corresponding revision to the strong claim and added the discussion regarding encoded 3D information in the revised manuscript. This addition highlights the potential for our approach to extend beyond two-dimensional (2D) structures and encompass intricate three-dimensional (3D) information, such as 3D polarization structures of metasurfaces. We appreciate your feedback, which has enhanced the comprehensiveness of our work.

In the main text:

(Page 11, Line 308) Furthermore, the micro/nanostructures we have created within the hydrogels currently contain only two-dimensional (2D) multi-information. Nevertheless, we envision that our approach can be extended to fabricate intricate structures that include 3D information, such as 3D polarization structures of metasurfaces[33], and this expansion will provide exciting possibilities for achieving even more sophisticated functionalities and applications.

(Page 11, Line 315) This opens up captivating possibilities for dynamic and reconfigurable functionalities offered by various functional materials.

Comment #6:

Additional remarks:

A: Certain sections should be backed up by references, for example the photobleaching of dyes.

B: It would be good to clarify what is the key parameter for each of the effects: dose, fluence, flux, or others.

C: Could the authors provide more information on the mechanism of the induced changes (hot electrons vs. heat)?

→We appreciate the reviewer for the constructive suggestions. For Comment #6-A, we have added references to relevant sections, and please refer to Page 8, Line 216; Page 6, Line 180; Page 5, Line 144.

For the reply to Comment #6-B and C, it would be beneficial to understand the process of the fs laser enabled ablation. Multi-photon ablation using femtosecond lasers involves the precise removal of material from a target by delivering ultra-short laser pulses with high peak intensities. When these pulses interact with the materials, multiple photons are simultaneously absorbed by electrons in the material due to the high intensity, even though each photon's energy is typically below the material's bandgap. This multi-photon absorption results in electron excitation or ionization, initiating a cascade of events including rapid electron thermalization (**hot electrons**) and energy transfer to the material lattice. The localized **heating** causes material vaporization or sublimation, forming a plasma plume and ultimately leading to ablation. The process hinges on exceeding an ablation threshold, determined by the material's intrinsic properties and laser parameters (fluence, the energy delivered per unit area).

For photobleaching process, it is a process observed in fluorescence-based techniques where the fluorescence intensity of a fluorophore diminishes over time when exposed to light. Initially, photons are absorbed, exciting electrons to higher energy states. After a brief period of time, the electrons return to their ground state, emitting fluorescence. However, continuous or intense illumination can lead to photodamage.

This damage can manifest as chemical changes, the formation of reactive species, or irreversible transitions between energy states, rendering the fluorophore unable to emit fluorescence.

In summary, for Comment #6-B, the fluence of the fs determines the ablation threshold (Page 4, Line 93), and this parameter is a critical factor in determining whether ablation will occur. Above the threshold, the photons provide enough energy to break the bonds within the material, leading to ablation and the removal of material. For Comment #6-C, the induced changes for ablation is the result of both hot electrons and induced localized heat, while that for photobleaching is due to direct chemical changes by adsorption of photons rather than the hot electrons and induced heat, even though they play a role in some photodamage process.

Reviewer #2:

Summary

In this paper, the authors have detailed their work on multi-information writing in a hydrogel, LIHAMS, utilizing wavelength-dependent diffraction of ablated grooves, its polarization sensitivity, and photobleaching of fluorescent dyes. I am impressed by the careful optimization of laser irradiation conditions for each optical mode to inscribe information in xy dimensions, culminating in the successful recording of different information on a plate. The authors have adeptly integrated well-established principles of recording, physics, and photochemistry behind the technology with the material they developed. The manuscript is well articulated, and the technical demonstrations appear sound. Moreover, the experiments seem well-executed, as I could not identify any flaws in the procedures. However, while the authors have skillfully brought together existing knowledge and their own material development, it is challenging to discern novel scientific findings in the current manuscript. It would enhance the paper significantly if the authors could elucidate what made this achievement possible, perhaps from a materials perspective, and highlight any novel scientific findings. Additionally, strengthening the introduction to appeal to a broader audience, as targeted by this journal, would be beneficial. With these improvements, I can recommend the publication of this paper in Nature Communications. To facilitate better understanding of the research for readers, I suggest the inclusion of the following points:

→We appreciate the reviewer's positive opinion about our work. In response to the insightful comments from the reviewer, we conducted additional experiments and analyses to further underscore the novelty and significance of our study. According to the reviewer's constructive suggestions, we have thoroughly revised the abstract and introduction sections, and believe its quality has been significantly improved.

Comment #1:

Detailed exposition of exposure conditions (intensity, scanning speed, lens specifications for writing and reading, etc.) for each experimental demonstration.

→We appreciate for the reviewer's constructive suggestion. Accordingly, we have provided detailed exposure conditions of the fs laser and information of the objective we used in the revised manuscript.

In the main text:

(Page 13, Line 414) The LIHAM film on the glass substrate was then directly written through the fs laser (centre wavelength: 780 nm, pulse duration: 80 fs, repetition rate: 80 MHz, average output power (scanning

power): 0-50 mW) with programmable dosage domains and writing processes (Nanoscribe, GmbH). We used a 63X immersion objective (NA=1.4, focal Width (FWD)=0.19 mm, parfocal Length=45.06 mm, Zeiss) to enable the micro/nanofabrication within the LIHAM film.

(Page 14, Line 428) The scanning speed is set at 1500 µm/s for writing image 1 and 2; the scanning speed and power are set at 100,000 µm/s and 30 mW for writing image 3.

(Page 18, Line 520) The scanning speed is set at 1000 µm/s, while the scanning power varies within a range of 30-50 mW at different locations within the written patterns.

(Page 19, Line 537) The scanning speed and power are set at 1000 µm/s and 50 mW for writing the above samples.

(Page 20, Line 549) The scanning speed and power are set at 100,000 µm/s and 50 mW for writing the above samples.

Comment #2:

A discussion on the capability of recording information in 3D space (x,y,z) using all modalities together.

I believe that addressing these points will provide a more comprehensive perspective of the research and render the manuscript more impactful for the broad readership of Nature Communications.

→We appreciate the reviewer for the constructive suggestion. Currently, our approach can be used created micro/nanostructures with 2D information, while we believe we could extend the fabrication to make intricate metasurfaces that encompass intricate three-dimensional (3D) information, such as 3D polarization structures of metasurfaces (*Light: Sci. Appl.*, 11, 302 (2022)). Accordingly, we have made corresponding revision and added the discussion regarding encoded 3D information in the revised manuscript to enhance the comprehensiveness of our work.

In the main text:

(Page 11, Line 308) Furthermore, the micro/nanostructures we have created within the hydrogels currently contain only two-dimensional (2D) multi-information. Nevertheless, we envision that our approach can be extended to fabricate intricate structures that include 3D information, such as 3D polarization structures of metasurfaces[33], and this expansion will provide exciting possibilities for achieving even more sophisticated functionalities and applications.

Reviewer #3:

Summary

This manuscript describes a laser processing method that combines three laser 2D patterning techniques to write different micro-structures on hydrogel surface, and to produce images based on structure color, polarization state, and fluorescent intensity. Visually impressive images were produced. However, this work is a combination of three well, long-established methods. It lacks the novelty or innovation in terms of advancing the laser processing method (or enhancing the fundamental understanding of laser processes). Hence it is not suitable for publication in Nature Comm. Laser micromachining of sub-micrometer structures, grating like structures (including the laser-induced periodic surface structures or LIPSS) for displaying different colors, polarization dependent microstructures, and direct laser write of fluorescent microstructures all have been investigated and well-established for tens of years. (Also see below for the specific comments.) Additionally, the application of this work seems to be limited. In addition to those loosely mentioned at the beginning of the abstract, the only other relevant use has to do with the specific material in the author's group in Line 86-88. Even there, it is not clear how the fabricated structures will benefit LIHAMS. Therefore, the impact of this work is limited. Here are some additional comments:

→We appreciate the meticulous evaluation provided by the reviewer concerning our manuscript. The constructive feedback we received has played a pivotal role in elevating the overall quality and impact of our research. In response to the insightful comments from the reviewer, we conducted additional experiments and analyses to further underscore the novelty and significance of our study. As a result, we believe that our work now constitutes an important and valuable contribution to our field of study. To enhance the clarity of our novel concept and strengthen the innovation inherent in our work, we have summarized the innovative aspects into the following **key points (1-3)**:

(1) Our work introduces **a general strategy for the micro/nanofabrication of hydrogels**. Noted by their high content of water (typically exceeding 90% by weight), hydrogels are recognized for **their inherent softness**. While the softness of hydrogels offers unique advantages, such as tissue-compatibility, it also poses **significant challenges when it comes to creating micro/nanostructures within them.** Micro/nanostructures produced within hydrogels using various fs laser-based techniques, such as two-photon polymerization of hydrogel precursors, hydrogel molding, and direct laser ablation, often **lack the sufficient self-support, leading to structural instability and collapse.** Additionally, the **thermal effects** induced by fs lasers inevitably result in **undesirable deformations in hydrogels**, stemming from either the hydrogels'

thermoresponsive properties or the laser heating-induced bubbles, thereby introducing an additional layer of **complexity to the precise fabrication** of hydrogel structures. In response, we have proposed a strategy by **dehydrating hydrogels**, enabling **high-precision manufacturing of hydrogel materials**. Dehydration not only **reinforces the self-support** of fabricated structures (the modulus enhances more than three orders of magnitude), but also temporarily **arrest or "freeze" their dynamic behaviors** during the laser fabrication process. To illustrate the efficacy of our strategy, we have applied it to a recently developed thermoresponsive hydrogel by our group, successfully achieving the **encoding of multi-information within the hydrogels.**

(2) Our approach yields microstructures with the remarkable capability **to simultaneously display multiple independent sets of information at the same location within hydrogels**, achieved through the use of **versatile dosage domains of fs laser**. While previous research has individually explored fs-laser micromachining to create intricate structures capable of exhibiting various characteristics such as different colors, polarization-dependent microstructures, and direct laser writing of fluorescent patterns, **the integration of these multi-channel information displays** at a single location using versatile laser dosage **has been a rare occurrence**. Through systematic design and optimization for each distinct channel within the same location, achieved by rationally applying different fs laser dosages, we have successfully achieved their **independent displays with minimal cross-talk effects**.

(3) Our thermoresponsive hydrogel, embedded with encoded multi-information, offers **a means of stimuli-responsive manipulation of the encoded information**. The dehydrated hydrogels can be restored to their original state (a water content exceeding 90%) through rehydrating inside water. As these compact hydrophilic polymer networks regain their original porous network structure upon absorbing water, the **thermoresponsive behavior recovers**. Given that the multi-channel information is sensitive to the geometric parameters, such as the height of the as-fabricated structures, **thermal alterations** in these geometries enable **dynamic changes in the displayed information**. For instance, raising the temperature of the hydrogel **sequentially displays different images across different encoded channels**.

Consequently, we have made substantial revisions to our original manuscript, emphasizing these key novelty points. **We have also conducted new experiments, redesigned our main figures, and rewritten the introduction section of the manuscript.** Through this clarification of the novelties, we believe that our work represents a meaningful contribution to the field.

In the main text:

(Page 1, Line 13) Creating micro/nanostructures containing multi-channel information within responsive hydrogels presents exciting opportunities for dynamically changing functionalities. However, fabricating these structures is immensely challenging due to the soft and dynamic nature of hydrogels, often resulting in unintended structural deformations or destruction. Here, we demonstrate that dehydrated hydrogels, treated by a programmable femtosecond laser, can allow for a robust fabrication of micro/nanostructures. The dehydration enhances rigidity of the hydrogels and temporarily locks the dynamic behaviors, significantly promoting their structural integrity during the fabrication process.

(Page 2, Line 32) Micro/nanofabrication techniques have garnered significant attention across various fields, including integrated electronics, optics[1,2], miniature robotics[3-6], and microelectromechanical systems (MEMS)[7]. Among the tools employed for fabricating tiny structures[8,9], femtosecond (fs) lasers stand out as highly efficient, versatile and extensively utilized strategies[10,11]. The distinguishing feature of fs lasers lies in their ability to deliver short pulse durations with exceptionally high peak intensity[12,13]. This characteristic facilitates precise energy deposition while minimizing heat transfer to the surrounding areas[14]. As a result, fs lasers empower the creation of high-precision structures across a wide spectrum of materials[11]. The versatility inherent in the photon-matter interactions achievable with fs lasers confers distinct advantages in micro/nanofabrication[12,15-17]. Processes such as photobleaching[17], two-photon polymerization[11], and multi-photon ablation[13] enable tailored material modifications, substantially expanding the fabrication toolbox. Therefore, exploration and utilization of the diverse dosage domains offered by fs lasers opens up new possibilities in fabrication, presenting exciting opportunities for the integration of miniature devices across many applications.

However, despite the versatility of fs laser-based fabrication processes across a diverse range of materials, the creation of hydrogel micro/nanostructures poses a challenge[18,19]. Hydrogels, composed of three-dimensional (3D) hydrophilic polymer networks containing a high-water content (typically more than 90% by weight), have gained widespread attention owing to their versatile and easily tunable properties[20-22]. Nonetheless, the inherent softness of hydrogel materials, often characterized by low stiffness (typically on the order of kPa), presents significant hurdles in their high-precision fabrication[23,24]. The micro/nanostructures created within hydrogels often lack sufficient self-support, leading to structural instability and collapse[25]. Moreover, the inevitably generated thermal effects induced by the laser contribute to unwanted deformations of hydrogels, which arise either from the hydrogels' thermoresponsive properties or the laser heating-induced bubbles, adding another layer of complexity to the precise fabrication of hydrogel structures[26,27]. These challenges compromise structural integrity, imposing formidable obstacles in achieving high-precision

structures within hydrogels, restricting the potential applications of miniature devices fabricated from hydrogels.

Here, we introduce a general strategy that allows for the micro/nanofabrication within soft and dynamic hydrogels by dehydrating the hydrogel. The dehydration of the hydrogel does not only bolster self-support of the fabricated structures but also transiently freezes their dynamic behaviors during the fabrication process. The versatility offered by various dosage domains of a fs laser empowers the creation of structures that can concurrently encompass multi-channel information within the responsive hydrogel, and exhibit multiple independent information at the same location on the hydrogel. Furthermore, we illustrate the dynamic capability of these structures in displaying intricate images sequentially through thermal activation of the hydrogel.

(Page 3, Line 72) **Micro/nanofabrication of multi-information within active hydrogels**

While the versatility offered by fs lasers in enabling different photon-material interactions (**Fig. 1a**), the fabrication of intricate structures in soft hydrogels remains a formidable challenge. Hydrogel materials are known for their inherent softness (on the order of kPa). While the softness of hydrogels offers unique advantages, it also presents significant hurdles when it comes to creating micro/nanostructures. Numerous strategies have been attempted for micro/nanofabrication of hydrogels using fs lasers, including two-photon polymerization of hydrogel precursors (Fig. 1b and Supplementary Fig. 1a), hydrogel molding (Supplementary Fig. 1b), and direct laser ablation of hydrogels (Supplementary Fig. 1c). Unfortunately, these approaches have fallen short in achieving high-precision micro/nanostructures with high structural integrity in hydrogels (see detailed discussion in Supplementary Note 1). We proposed a method to bolster their mechanical rigidity   that relies on a reversible dehydration process of hydrogels (Fig. 1c and Supplementary Fig. 2). This process results in the compaction of the original porous network within the hydrogel, rendering a substantial enhancement in mechanical properties. Furthermore, the dynamic behavior of hydrogels can be temporarily arrested or "frozen", contributing to an augmented structural integrity during the fs laser fabrication process. These dehydrated hydrogels with intricately fabricated structures by fs laser, can recover their original porous network (high water content) and stimuli-response through rehydrating in water.

(Page 10, Line 290) While the softness of hydrogels offers distinct advantages, it also poses significant challenges when it comes to creating micro/nanostructures. In our work, a general strategy of hydrogel dehydration has been implemented before fs laser fabrication, which significantly increases the structural integrity of the resulting micro/nanostructures.

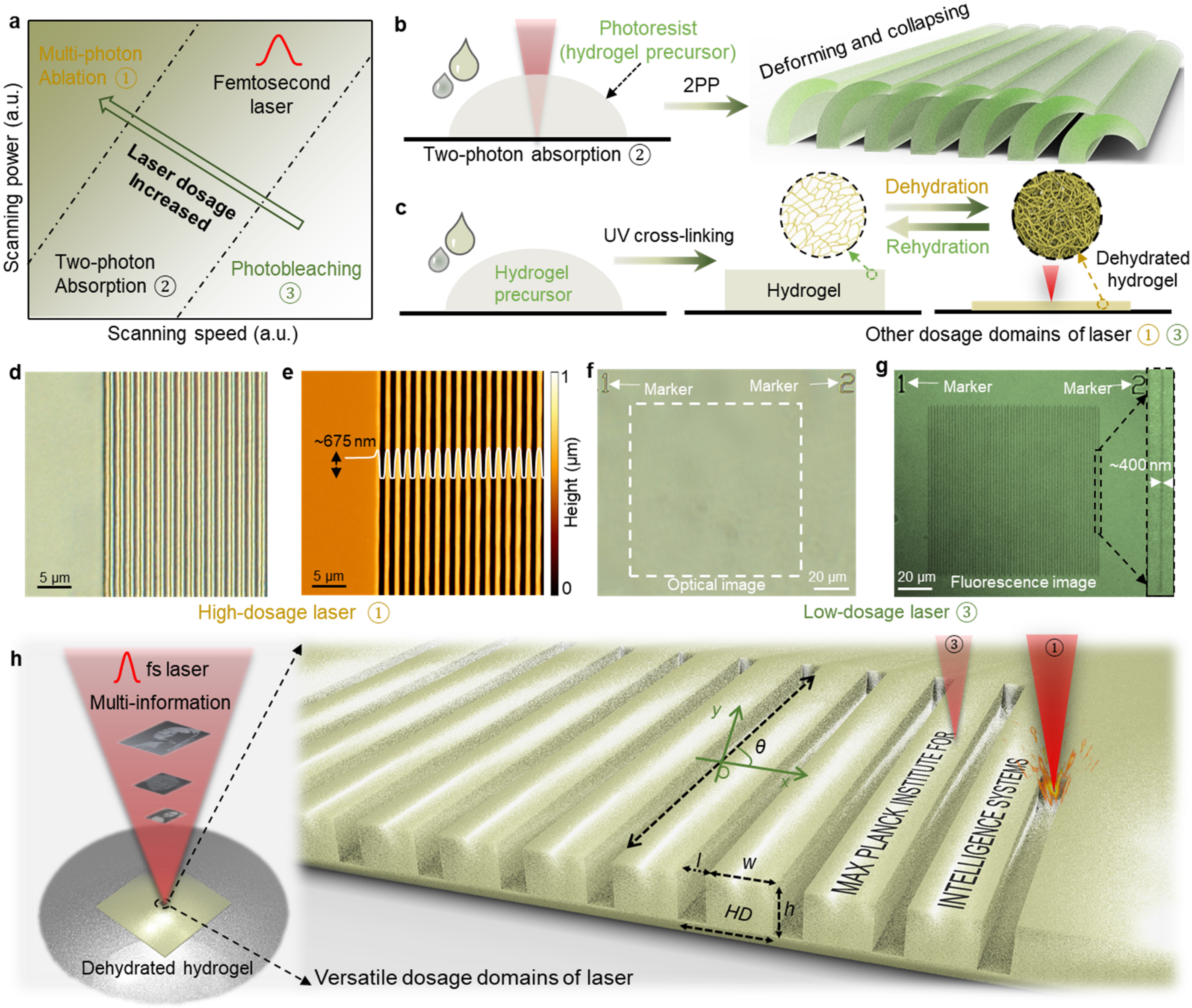

(Page 16, Line 483) **Fig. 1 Dehydration of thermoresponsive hydrogels enabling their high-precision fabrication for encoding multi-information using multi-dosage of the femtosecond (fs) laser. a**, Versatile dosage domains of the fs laser and their different uses in the photon-material interactions. **b**, Schematics showing the two-photon polymerized hydrogels are prone to deform or collapse due to the low rigidity of hydrogels. **c**, Schematic showing the dehydrated hydrogels enhance the structural integrity during their fs laser fabrication. **d**, Optical image depicting the grooves on a hydrogel surface fabricated using laser with a high-dosage domain. **e**, Atomic force microscopy (AFM) image showing these written grooves on the hydrogel surface. **f**, Optical images illustrating a hydrogel surface using a low-dosage domain. The numbers "*1*" and "*2*" are abated as markers. **g**, Confocal fluorescence microscopy image with hydrogel surface treated in the low-dosage domain. **h**, Schematics of the micro/nanofabrication of the dehydrated hydrogel film with encoded with multi-information.

In the Supplementary Information:

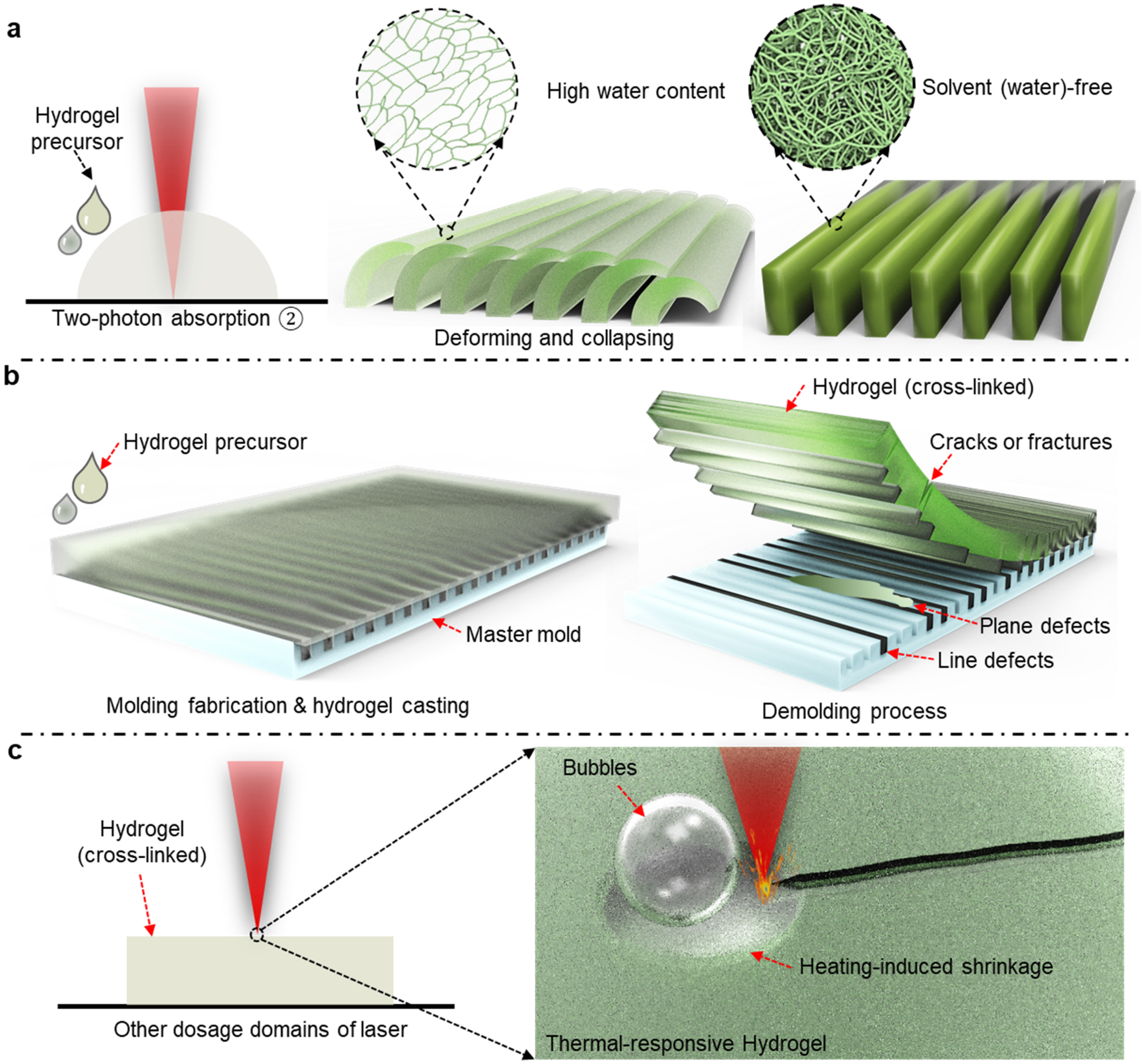

(Page 3, Line 79) **Supplementary Fig. 1 Schematic illustration showing the challenge of high-precision fabrication for hydrogel materials. a**, Schematics of two-photo polymerization of hydrogels, including high water content and water-free recipes. **b,** Schematics of the molding and demolding process of hydrogels for replicating intricate structures. **c,** Schematic of direct laser-ablated hydrogel.

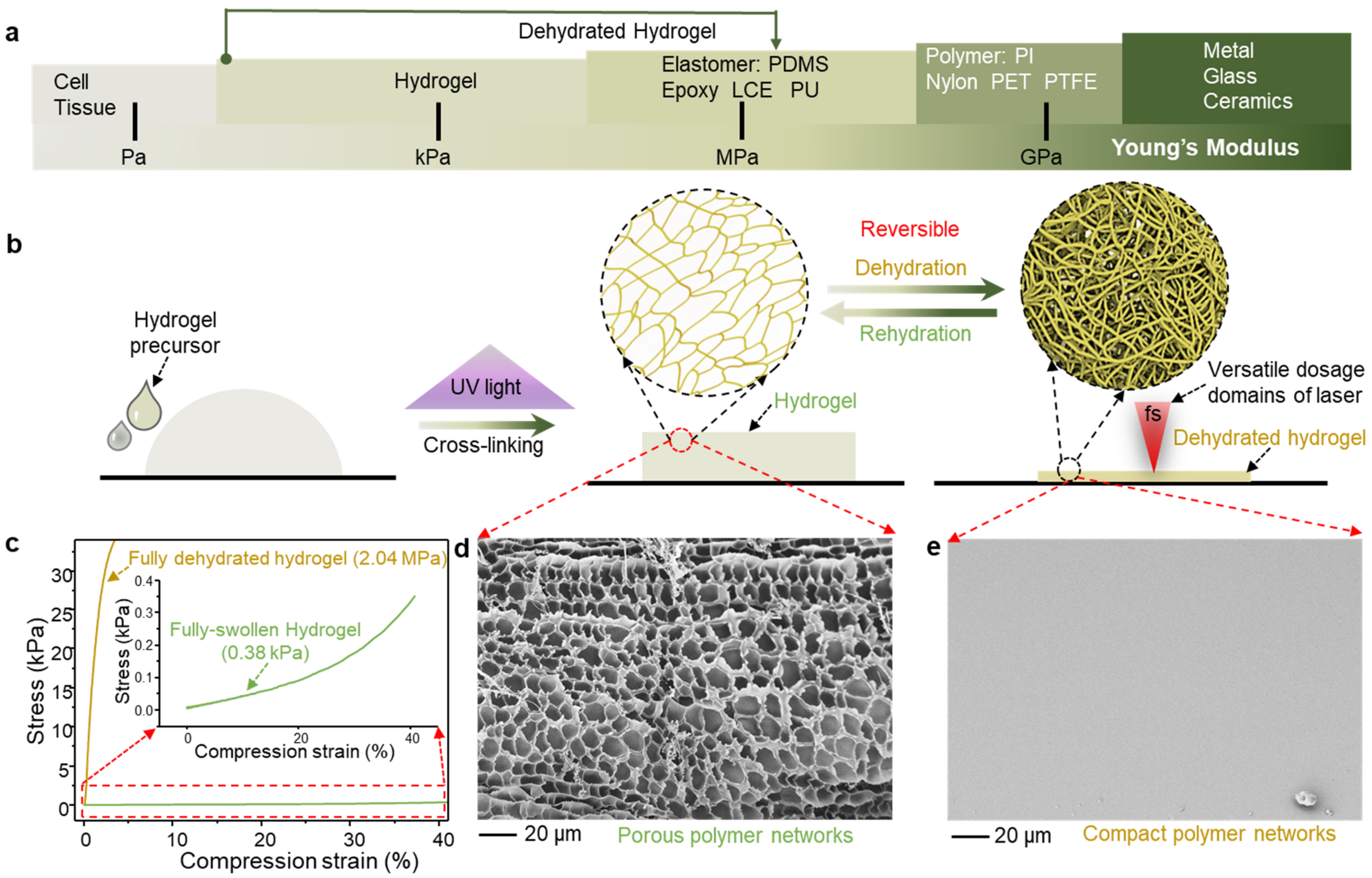

(Page 5, Line 86) **Supplementary Fig. 2 Dehydration of LIHAM Hydrogels enhances their structural integrity of the fabricated micro/nanostructures. a**, Comparison of the mechanical properties of hydrogels with versatile common materials. **b**, Schematic illustration of micro/nanofabrication process of the LIHAM hydrogels. **c**, Compression test of the LIHAM hydrogel and the dehyrated hyrogel. **d**, Scanning electron microscopy (SEM) images of LIHAM hydrogels, showing the porous mophologies. **e**, SEM image of dehydrated LIHAM hydrogel, showing a smooth morphologies as a result of the closely compact polymer networks after dehydration.

(Page 2, Line 19) **Supplementary Note 1. Typical stragies using femtosecond laser for the micro- and nanofabrication of hydrogel materials**

Femtosecond (fs) lasers have emerged as a highly efficient and versatile strategy for fabricating micro- and nanostructures across a wide range of materials[1]. This technique leverages versatile photon-matter interactions and encompasses both additive and subtractive manufacturing approaches.

Additive Manufacturing: One notable additive manufacturing approach for creating high-precision hydrogels is known as two-photon polymerization (2PP)[2,3]. In this process, a hydrogel precursor containing dispersed monomers in a solvent, typically water, undergoes site-specific crosslinking through two-photon absorption induced by the fs laser. This results in the creation of high-precision 3D structures at submicron

scales (see Supplementary Fig. 1a). However, hydrogel structures produced through 2PP, especially those with high aspect ratios, are susceptible to deformation or collapse due to their low stiffness[4]. This is primarily a consequence of their high water content (exceeding 90%). While solvent-free hydrogel precursors can improve structural integrity due to their stiff 3D polymer structures[5], this also leads to much higher cross-linking density of networks. As a consequence, the later water absorption is limited due to the high cross-linking density, resulting in a departure from typical hydrogel properties like softness and responsiveness based on water adsorption/desorption, which are permanently compromised.

Another additive manufacturing strategy involves molding precision structures from a 2PP-fabricated master mold[6]. Hydrogel precursor solution is cast onto the master mold, cross-linked with UV light, and then detached (Supplementary Fig. 1b). However, this method is also hampered by the softness of hydrogel materials, leading to problems like line/plane defects and cracks during demolding process due to the fragile and soft nature of hydrogels.

Subtractive Manufacturing: Subtractive manufacturing techniques involve directly exposing materials to fs laser ablation, allowing for the creation of high-precision structures. Nevertheless, the high water content and dynamic behavior of hydrogels pose significant challenges to achieving precision in this process. Laser-induced heating leads to intense water evaporation (generating disturbing bubbles) within the hydrogel polymer networks, causing deformation and defects during ablation. Additionally, certain hydrogels, such as thermally responsive variants, are sensitive to temperature changes, further undermining structural integrity.

Our Dehydration-Based Strategy: As structures made of most commercial photoresits are usually rigid (usually with moduli on the order of GPa), they can achieve high-precision farbication. Therefore, we believe that as materials become increasingly rigid, the fabricated high-precision structures tend to become more self-supporting, thereby enhancing the structural integrity of the resulting micro/nanostructures (Supplementary Fig. 2a). Therefore, it can be advantageous to improve their mechanical properties by dehydrating the hydrogels before laser processing, as illustrated in Supplementary Fig. 2b. The dehydration process serves a dual purpose: it not only enhances the mechanical properties (Young's modulus) of hydrogels more than three orders of magnitude (Supplementary Fig. 2c), but also temporarily stabilizes their dynamic behavior. The reason behind this effect lies in the transformation of the porous 3D network of hydrogels into a more compact structure after dehydration (Supplementary Figs. 2d and 2e). The stabilization is particularly beneficial for responsive hydrogels, such as the one we have recently developed (LIHAM)[7]. As the thermal response of these hydrogels is governed by the desorption/adsorption of water, the dehydration process (absence of water) can effectively arrest or freeze their thermal response.

Importantly, this dehydration does not alter the cross-linking density of the hydrogel. As a result, the dehydrated hydrogel can recover its high water content after micro/nanofabrication, thus retaining its thermal responsiveness. In this way, this reversible dehydration/rehydration process enables dynamic and reconfigurable functionalities in the fabricated structures. Our strategy offers a solution to the challenges associated with precision hydrogel manufacturing using fs lasers, opening up new possibilities for the dynamic and responsive design of hydrogel-based miniature structures and devices.

Comment #1:

Title: “all-in-one” needs to be changed. There are other features or structures that this method does not create, such as true 3D structures (also see below).

→We thank the reviewer for the kind suggestion. Accordingly, we have changed the previous title with “**Micro- and nanofabrication of dynamic hydrogels with multi-channel information**”.

Comment #2:

Abstract and introduction part of the main text: The topic of this manuscript is very much different from two-photon printing, as it does not deal with true 3D printing at all. Comparison to 3D printing should be kept to a minimum, while many similar printing/laser processing methods (fabrication of microscale or sub-microscale grooves, fabricating of grating like periodic structures including LIPSS, etc.) should be discussed and referenced in abstract, intro, and other places. For example, Line 36, 3D data storage using TPP or direct laser writing was presented as early as 1989. Encryption using DLW has also been extensively reported as a plausible application for a long time. Combination of subtraction and addition has also been studied extensively. Literature on these topics should be reviewed and included, in reference to the novelty of the current work.

→We appreciate the constructive suggestion and fully agree with the reviewer's assessment that the emphasis on two-photon polymerization in our previous manuscript was inappropriate. In response to the reviewer's feedback, we have undertaken substantial revisions to the abstract and introduction sections of the main text in the revised manuscript. Please refer to our detailed response in the "**Reply to the Summary**" for further details on these revisions.

Comment #3:

Line 21 should be “within the rest of the unablated areas”

→We thank the reviewer for the kind suggestion, and had made corresponding changes in the revised manuscript.

In the main text:

(Page 1, Line 21) By utilizing versatile dosage domains of the femtosecond laser, we create micro-grooves on the hydrogel surface through the use of a high-dosage mode, while also altering the fluorescent intensity within the rest of the non-ablated areas *via* a low-dosage laser.

Comment #4:

Line 22 should be “three-channels of information” or “three-channel information”

→We thank the reviewer for the kind suggestion, and had made corresponding changes in the revised manuscript.

In the main text:

(Page 1, Line 23) In this way, we rationally design a pixel unit containing three-channel information: structural color, polarization state, and fluorescent intensity, and encode three complex image information sets into these channels.

Comment #5:

Line 52, 243, 364, etc., the word “Besides” is used incorrectly and should be replaced with something like “Additionally”.

→We thank the reviewer for the kind suggestion, and have made corresponding changes in the revised manuscript (please refer to **Line 255, 405**).

Comment #6:

The section starting from Line 67: in the description of the all-in-one lithography process, it seems the two-photon absorption region, or region 2 in Figure 1A is left unused. This should be addressed since discussion elsewhere in the text suggests region 2 is useful to this process.

→We appreciate the constructive suggestion. Indeed, we acknowledge that the term "all-in-one" in our original manuscript may have been misleading. In response to this feedback, we have made appropriate

revisions in the revised manuscript by refraining from using this concept and minimizing the discussion of the previously irrelevant focus on the two-photon absorption region.

In the main text:

(Page 2, Line 32) Micro/nanofabrication techniques have garnered significant attention across various fields, including integrated electronics, optics[1,2], miniature robotics[3-6], and microelectromechanical systems (MEMS)[7]. Among the tools employed for fabricating tiny structures[8,9], femtosecond (fs) lasers stand out as highly efficient, versatile and extensively utilized strategies[10,11]. The distinguishing feature of fs lasers lies in their ability to deliver short pulse durations with exceptionally high peak intensity[12,13]. This characteristic facilitates precise energy deposition while minimizing heat transfer to the surrounding areas[14]. As a result, fs lasers empower the creation of high-precision structures across a wide spectrum of materials[11]. The versatility inherent in the photon-matter interactions achievable with fs lasers confers distinct advantages in micro/nanofabrication[12,15-17]. Processes such as photobleaching[17], two-photon polymerization[11], and multi-photon ablation[13] enable tailored material modifications, substantially expanding the fabrication toolbox. Therefore, exploration and utilization of the diverse dosage domains offered by fs lasers opens up new possibilities in fabrication, presenting exciting opportunities for the integration of miniature devices across many applications.

(Page 3, Line 72) **Micro/nanofabrication of multi-information within active hydrogels**

While the versatility offered by fs lasers in enabling different photon-material interactions (**Fig. 1a**), the fabrication of intricate structures in soft hydrogels remains a formidable challenge. Hydrogel materials are known for their inherent softness (on the order of kPa). While the softness of hydrogels offers unique advantages, it also presents significant hurdles when it comes to creating micro/nanostructures. Numerous strategies have been attempted for micro/nanofabrication of hydrogels using fs lasers, including two-photon polymerization of hydrogel precursors (Fig. 1b and Supplementary Fig. 1a), hydrogel molding (Supplementary Fig. 1b), and direct laser ablation of hydrogels (Supplementary Fig. 1c). Unfortunately, these approaches have fallen short in achieving high-precision micro/nanostructures with high structural integrity in hydrogels (see detailed discussion in Supplementary Note 1). We proposed a method to bolster their mechanical rigidity that relies on a reversible dehydration process of hydrogels (Fig. 1c and Supplementary Fig. 2). This process results in the compaction of the original porous network within the hydrogel, rendering a substantial enhancement in mechanical properties. Furthermore, the dynamic behavior of hydrogels can be temporarily arrested or "frozen", contributing to an augmented structural integrity during the fs laser

fabrication process. These dehydrated hydrogels with intricately fabricated structures by fs laser, can recover their original porous network (high water content) and stimuli-response through rehydrating in water.

Comment #7:

Furthermore, line 109 suggests the two-photon absorption region should be avoided all together. This again shows it is unnecessary to provide extensive discussions in abstract and introduction of two-photon processes. Also, it would be better to clarify in the introduction, ~line 43, that the typical two-photon absorption regimes for cross-linking based additive manufacturing are avoided and only multi-photon ablation regimes for subtractive manufacturing are used.

→We appreciate the constructive suggestion and concur with the reviewer's assessment that the prominence given to two-photon polymerization in our initial manuscript was inappropriate. In response to the reviewer's valuable feedback, we have undertaken significant revisions to both the abstract and introduction sections within the revised manuscript. Please refer to our comprehensive response in the "**Reply to the Summary**" section for a more in-depth exploration of these revisions.

Comment #8:

Line 121, a minimum hatching distance of 1 um is relatively large compared to other lithography or laser direct write methods. Is this limitation attributed solely to the thermal dynamics of the hydrogel? If so, do the benefits of the thermal sensitivity of the LIHAM hydrogel offset the disadvantage of limiting the hatching distance?

→ We appreciate the reviewer for raising this important question. Indeed, the minimum hatching distance of 1 μm is primarily attributed to the soft nature of our hydrogel materials. While the dehydration process significantly enhances the rigidity of these hydrogels, transitioning from E=380 kPa for the swollen hydrogel to 2.04 MPa for the dehydrated hydrogel (Supplementary Fig. 2c), it's important to acknowledge that this enhanced modulus is still relatively low when compared to most commercial photoresist fabricated structures, such as IP-S (E=2.1 GPa), IP-Dip (E=1.3 GPa), IP-Q (E=3.1 GPa), IP-L (E=4.7 GPa), and IP-Q (E=3.4 GPa). The rigidity of printed structures is crucial for their preventing collapse or deformation, as structures made of rigid materials tend to self-support, ensuring high structural integrity.

However, despite the inherent softness of our dehydrated hydrogel, it's worth noting that we can achieve submicron precision (a few hundred nanometers), which is already comparable to other commercial rigid materials. Additionally, we have the flexibility to adjust laser parameters to tailor the morphologies of the

ablated grooves, including scanning power and speed. For instance, increasing the scanning speed results in a reduction in the full width at half maximum (FWHM) of the resulting groove, dropping from approximately 800 nm to 550 nm (as illustrated in Supplementary Fig. 5). This means that we can attain narrower grooves by using a lower dosage of laser, allowing us to achieve a hatching distance of less than 1 µm. We have incorporated these results into the revised manuscript for clarity and completeness.

In the main text:

(Page 5, Line 139) Modifying the dosage of laser by increasing the scanning power or reducing the scanning speed can tailor the morphologies of the obtained grooves and result in the creation of a deeper groove (Figs. 2e, f) and a broader full width at half maximum (FWHM) of the groove (Supplementary Fig. 5).

In the Supplementary Information:

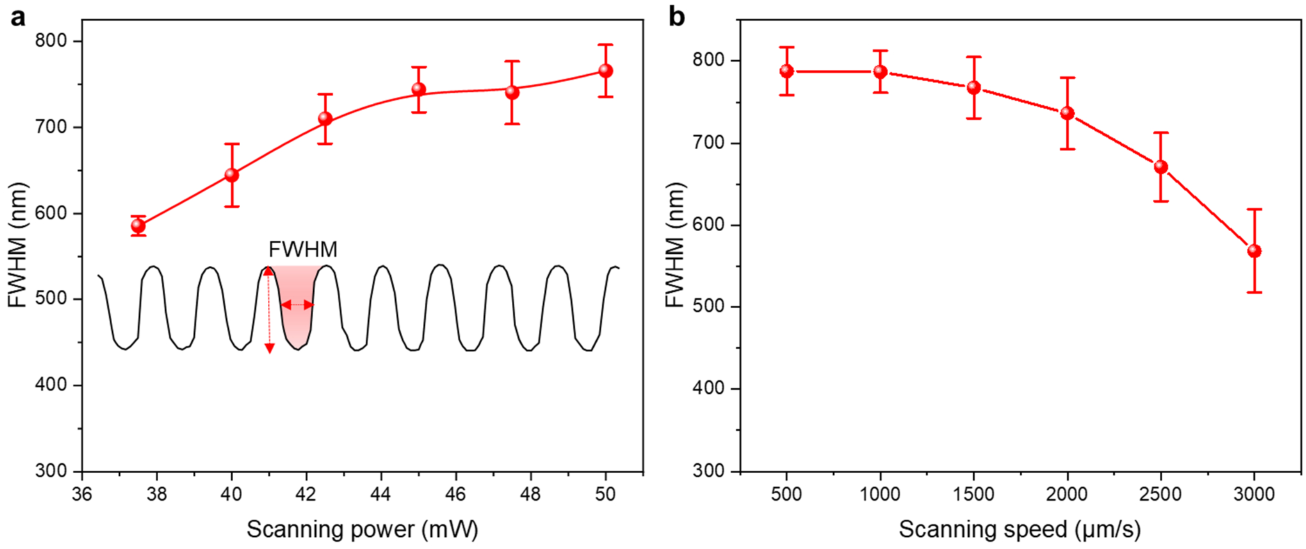

(Page 8, Line 108) **Supplementary Fig. 5 Effect of the scanning power (a) and speed (b) of the laser on the full width at half maximum (FWHM) of the obtained grooves.**

Comment #9:

Line 220, why does larger HD increase dosage of the laser? Are the authors implying these two are disconnected and larger HD requires more dosage be used? Otherwise, larger HD should decrease the dosage of the laser in a given volume when all else is constant.

→We appreciate the reviewer for bringing this matter to our attention. Upon thorough review, we have identified a source of confusion regarding the terms involving the hatching distance (*HD*) and the spacing between two adjacent hatching lines. In Figures 5E and 5F, our intention is to convey that as the *HD*

decreases, more untreated area will be photobleached, resulting in a substantial decrease in their overall fluorescent retention. We have accordingly made the necessary correction in the revised manuscript.

In the main text:

(Page 8, Line 231) Additionally, a decreased *HD* leads to an expansion of the photobleached area, ultimately causing a remarkable decrease in the overall fluorescent intensity (Figs. 5e, f).

Comment #10:

Figure 5E shows fluorescent retention increases as HD increase not the other way around as was stated in the text, Line 221.

→We appreciate the reviewer for bringing this matter to our attention, which aligns with the concerns raised in Comment #9. Please refer to our **Reply to Comment #9** for clarification on this issue.

Comment #11:

Line 277, dynamic switching between the image channels implies reversibility. Can all three channels of information be reobtained after the LIHAM hydrogel is heated/cooled? Is the Mona Lisa information in Figure 6K kept or lost after the heating is completed?

→We thank the reviewer for the question. The LIHAM hydrogel shows good reversibility along the changes of temperatures, and the information encoded within the hydrogel can be recovered. Upon heating, the LIHAM hydrogel undergoes deformation, resulting in a decrease in the heights of the groove structures (Supplementary Fig. 15). As the colorful information (*Mona Lisa*) is mainly determined by the heights, the heights of the groove structures recover as the temperature, thus reappearing the *Mona Lisa* information. As the thermal actuation process does not involve any irreversible alterations in the adsorption and desorption of water (please also refer to more details in our recent publication, *Nat. Mater*., 2023, 22, 1243-1252), the information change within the three channels is completely reversible. We added the discussion in the revised manuscript.

In the main text:

(Page 10, Line 286) Furthermore, it is worth noting that the thermal deformation of the LIHAM hydrogel is fully reversible[28], and the information encoded within the hydrogel is thus also re-emerge upon cooling.

Comment #12:

Outlook, Line 296: again, if this process is to be compared directly to 2PP the 2D and fully-subtractive aspect of this work must be acknowledged. Again, a significant advantage of 2PP is the ability to additively create true 3D sub-micro structures. This work only presents 2D structures.

→We appreciate the reviewer's constructive suggestion and agree that the extensive discussion of two-photon polymerization in our initial manuscript was not appropriate. In light of the reviewer's insightful feedback, we have made substantial revisions to the abstract and introduction sections in the revised manuscript. For a more detailed examination of these revisions, please refer to our comprehensive response in the "**Reply to the Summary**" section.

Comment #13:

Line 371, the impressive capabilities of the Nanoscribe were utilized, but many specifics of the Nanoscribe parameters are available to public. More details on the experimental setup should be provided so that this work can be repeated on any DLW experimental setup, i.e., laser specifications, optical setup (objective lens, dip-in or oil-immersion printing, and laser scanning hardware etc.

→We appreciate the reviewer's constructive suggestion. Accordingly, we have provided more detailed information in the revised manuscript.

In the main text:

(Page 13, Line 414) The dehydrated LIHAM film on the glass substrate was then directly written through the fs laser (center wavelength: 780 nm, pulse duration: 80 fs, repetition rate: 80 MHz, average output power (scanning power): 0-50 mW) with programmable dosage domains and writing processes (DeScribe software, Nanoscribe GmbH). We used a 63X oil-immersion objective (NA=1.4, focal width (FWD)=0.19 mm, parfocal length=45.06 mm, Zeiss) to enable the micro/nanofabrication within the LIHAM film.

(Page 14, Line 428) The scanning speed is set at 1500 μm/s for writing image 1 and 2; the scanning speed and power are set at 100,000 μm/s and 30 mW for writing image 3.

(Page 18, Line 520) The scanning speed is set at 1000 μm/s, while the scanning power varies within a range of 30-50 mW at different locations within the written patterns.

(Page 19, Line 537) The scanning speed and power are set at 1000 µm/s and 50 mW for writing the above samples.

(Page 20, Line 549) The scanning speed and power are set at 100,000 µm/s and 50 mW for writing the above samples

REVIEWERS' COMMENTS

Reviewer #1 (Remarks to the Author):

In authors have addressed all reviewer comments and described their revisions in detail. All small comments and suggestions for improvement have been addressed satisfactorily.

The doubts by reviewers 1 and 3 regarding the impact of the results and their novelty have been addressed extensively, however, not convincingly. Substantial modifications of the manuscript have been applied in response to these doubts and the third reviewer's concern regarding the core terminology ("all-in-one") and the focus on 2-photon-polymerization. In the revised version, large parts of the original motivation have been replaced by different aspects of micro-/nano-patterning. The novelty and impact are now based on the improvement of patterning by dehydration (and rehydration) of the hydrogel, which was not even mentioned in the main text originally. The outcome is a process- and materials-based improvement of 2D patterning, demonstrated with scientifically sound experiments. However, despite placing the work in a different context and substantially changing the aspects considered new, it is still a superposition of existing methods rather than a technological or scientific milestone.

In summary, the doubts related to the potential impact of the work raised by two reviewers have not been eliminated, and I do not recommend publication in Nature Communications.

Reviewer #2 (Remarks to the Author):

The authors revised the manuscript according to the reviewer's comments. The manuscript can be published in Nature Communications.

Response to Reviewers' comments

We appreciate the reviewers for their contribution on our manuscript. Please find our point-by-point responses to the reviewers' comments preceded by blue arrows (→) below. Additionally, we have listed all editorial format changes made in the main text of our manuscript and highlighted them in yellow.

Reviewer #1:

Summary

In authors have addressed all reviewer comments and described their revisions in detail. All small comments and suggestions for improvement have been addressed satisfactorily.

The doubts by reviewers 1 and 3 regarding the impact of the results and their novelty have been addressed extensively, however, not convincingly. Substantial modifications of the manuscript have been applied in response to these doubts and the third reviewer's concern regarding the core terminology ("all-in-one") and the focus on 2-photon-polymerization. In the revised version, large parts of the original motivation have been replaced by different aspects of micro-/nano-patterning. The novelty and impact are now based on the improvement of patterning by dehydration (and rehydration) of the hydrogel, which was not even mentioned in the main text originally. The outcome is a process- and materials-based improvement of 2D patterning, demonstrated with scientifically sound experiments. However, despite placing the work in a different context and substantially changing the aspects considered new, it is still a superposition of existing methods rather than a technological or scientific milestone.

In summary, the doubts related to the potential impact of the work raised by two reviewers have not been eliminated, and I do not recommend publication in Nature Communications.

→We sincerely thank the reviewer for their detailed feedback and acknowledge their concerns regarding the novelty and impact of our work. We appreciate the recognition that we have satisfactorily addressed all small comments and suggestions.

In revising our manuscript, we focused on clarifying the impact and novelty of our approach. We thoroughly revised our initial emphasis on the "all-in-one" concept and two-photon polymerization (2PP) to avoid misinterpretation about our work's scope and impact. Following the reviewer's advice, we reduced the emphasis on 2PP, given that our work does not involve the laser dosage domain of 2PP, to prevent confusion among readers.

We have addressed a significant challenge in the micro/nanofabrication of hydrogel materials, which arises from their high-water content, ultra-soft nature, and dynamic deforming properties. Our proposed solution involves the dehydration of hydrogels and the use of a multi-dosage domain of femtosecond (fs) lasers. Dehydrating hydrogels enhances their mechanical properties, allowing for robust, self-supporting fine structures, and temporarily locks their dynamic behaviors, thereby boosting structural integrity during fabrication.

While we agree that our work may not represent a technological or scientific *milestone*, we assert its relevance as a foundational step in micro-/nanofabrication of hydrogel materials. The strategy we propose for achieving high-precision hydrogel structures is not only innovative but also lays the groundwork for future exploration of their functionality advancements in this field, as we have primarily demonstrated the novel dynamic behaviors (automated displaying distinct image information) based on this high-precision fabrication. To summarize our innovations based on this strategy, we highlight three key aspects: 1) A General Strategy for Hydrogel Micro/Nanofabrication; 2) Multiple Independent Information Displays within Hydrogels; 3) Stimuli-Responsive Manipulation of Encoded Information. We hope that the clarifications provided adequately address the concerns and demonstrate the merit of our work for the publication.

Reviewer #2:

Summary

The authors revised the manuscript according to the reviewer's comments. The manuscript can be published in Nature Communications.

→We thank the reviewer for the time and efforts on our manuscript.
